# Spontaneous Hetero-attachment of Single-Component Colloidal Precursors for the Synthesis of Asymmetric Au–Ag_2_X (X = S, Se) Heterodimers

**DOI:** 10.1021/acs.chemmater.2c01838

**Published:** 2022-12-12

**Authors:** Mengxi Lin, Guillem Montana, Javier Blanco, Lluís Yedra, Heleen van Gog, Marijn A. van Huis, Miguel López-Haro, José Juan Calvino, Sònia Estradé, Francesca Peiró, Albert Figuerola

**Affiliations:** † Department of Inorganic and Organic Chemistry, Inorganic Chemistry Section, University of Barcelona, Carrer de Martí i Franquès, 1-11, 08028Barcelona, Spain; ‡ Institute of Nanoscience and Nanotechnology, University of Barcelona, Carrer de Martí i Franquès, 1-11, 08028Barcelona, Spain; § Laboratory of Electron Nanoscopies (LENS-MIND), Department of Electronics and Biomedical Engineering, Universitat de Barcelona, C/Martí I Franquès 1, 08028, Barcelona, Spain; ∥ Nanostructured Materials and Interfaces, Zernike Institute for Advanced Materials, University of Groningen, Nijenborgh 4, 9747 AGGroningen, Netherlands; ⊥ Soft Condensed Matter, Debye Institute for Nanomaterials Science, Utrecht University, Princetonplein 5, 3584 CCUtrecht, Netherlands; # Departamento de Ciencia de los Materiales e Ingeniería Metalúrgica y Química Inorgánica, Facultad de Ciencias, Universidad de Cádiz, Cádiz11510, Spain

## Abstract

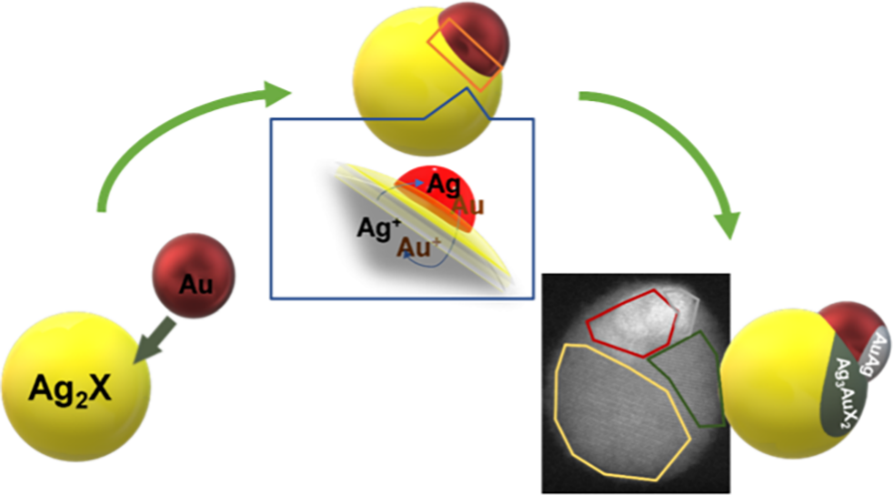

Finding simple, easily controlled, and flexible synthetic routes for the preparation of ternary and hybrid nanostructured semiconductors is always highly desirable, especially to fulfill the requirements for mass production to enable application to many fields such as optoelectronics, thermoelectricity, and catalysis. Moreover, understanding the underlying reaction mechanisms is equally important, offering a starting point for its extrapolation from one system to another. In this work, we developed a new and more straightforward colloidal synthetic way to form hybrid Au–Ag_2_X (X = S, Se) nanoparticles under mild conditions through the reaction of Au and Ag_2_X nanostructured precursors in solution. At the solid–solid interface between metallic domains and the binary chalcogenide domains, a small fraction of a ternary AuAg_3_X_2_ phase was observed to have grown as a consequence of a solid-state electrochemical reaction, as confirmed by computational studies. Thus, the formation of stable ternary phases drives the selective hetero-attachment of Au and Ag_2_X nanoparticles in solution, consolidates the interface between their domains, and stabilizes the whole hybrid Au–Ag_2_X systems.

Producing nanoscale heterostructures in which the distinct nanomaterial domains are coupled together through solid–solid interfaces enables multifunctionality in a single nanosystem.^1,2^ Beyond the size, shape, and composition-tunable optical, magnetic, electronic, and catalytic properties of single-component nanomaterials, the tunability of nanoscale heterostructures can be further expanded by controlling the interface, spatial arrangements, and configurations among different domains.^3^ The combined properties were also found to often surpass the functionality of individual components due to the synergistic effect.^4^ For instance, integrating CdS and MoS_2_ into one system not only enlarges the light absorption spectra in comparison to the individual components but also enhances the photochemical performance as photocatalysts.^5^ The transformation from uniform core@shell to nano-dumbbell Au–CdSe hybrids is another example illustrating the importance of the hybrid configuration and interface design, resulting in a wide optical response range across visible and near-infrared regions,^6^ which could not be achieved by simply mixing the two materials.^7^

In order to achieve such sophisticated colloidal heterostructured nanoparticles (NPs) with programmable features, the seed-mediated growth method is one of the most extensively used approaches whereby the pre-existing NPs serve as seeds and allow the subsequent nucleation and growth of another material directly on their surfaces.^8^ This approach opens the door to the synthesis and study of a large variety of hybrid systems.^9−12^ However, when it comes to the design of some specific hybrid NPs, this approach begins to show its flaws, giving rise to its vulnerability in the multiple-step process. In other words, the final product is highly influenced by numerous reaction parameters and some underlying mechanisms still remain unknown or they are still difficult to control.^13^ For example, the size and morphology of seed particles influence deeply the nucleation and growth processes of second domains, and on the other hand, the seeds themselves are very sensitive to the reaction conditions such as temperature, surfactants, and solvent.^13,14^

Given all these challenges, alternative methods for synthesis of complex hybrid NPs are highly desirable so that the variety of synthetic tools could be further expanded to fulfill the needs of the scientific community. Innovative synthetic routes proposed in the past years suggest the use of pre-made nanoparticles as precursor alternatives for the synthesis of further nanostructures in such a way that the final products are obtained simply by the attachment of pre-synthesized NPs in solution (or by their transformation) and new solid domains do not need to nucleate.^15,16^ Soft reaction conditions are often required in these cases compared to traditional solid-state chemistry, considering the increased reactivity of nanoparticles with high surface-to-volume ratios and short atomic diffusion distances due to their small dimensions. Additionally, compared to standard bottom-up wet methods, the use of nanoparticles as starting materials often avoids both (1) the need for significant amounts of surfactant molecules in the solution acting as stabilizers and size and shape-driving agents and (2) the presence of metallic counteranions that might interfere in the reaction as well as compromise the physical performance of the final material.

Cation exchange (CE) reactions in solution are one example of these new types of synthetic approaches. This is one of the most useful post-synthetic transformations, and it has been widely applied for preparing heterostructured NPs, resulting in good preservation of the morphology of initial materials while the compositions are modified.^8,17−20^ In a CE reaction, a material acts as a host lattice and permits the exchange reaction between its own cations and other guest cations. Over the last years, several new and compositionally complex NPs have been successfully obtained, such as Cu_5_FeS_4_/Cu_2–*x*_S/Cu_5_FeS_4_ nanosandwiches with exciting physicochemical properties,^21^ (CuGaIn)S_2_ nanocrystals (NCs) with 10-fold higher photoluminescence quantum yields compared to their parental nanostructures,^22^ and various metal-doped perovskite quantum dots.^23,24^

In the previous publications of our group, gradual cation exchange reactions between gold chloride and silver chalcogenide NPs have been studied.^25,26^ The partial exchange of Ag^+^ by Au^+^ cations in the silver chalcogenide lattice entails the formation of ternary phases, a process that is kinetically favored, and thus it occurs fast at room temperature and without the need for additional ligands except for those required to solubilize the gold molecular precursor and stabilize the silver chalcogenide NPs in organic apolar solvents. The easiness of this cation exchange reaction suggests a high stability for the ternary products formed.

The high affinity between gold and silver chalcogenide lattices observed in our previous works made us consider one further step: here, we describe the reaction of solely nanostructured materials, that is, Au NPs and Ag_2_X (X = S, Se) NPs, leading to heterostructured or hybrid Au–Ag_2_X NPs. The reactions occur at room temperature and atmospheric pressure in solution without the addition of surfactants or molecular precursors. Experimental data and calculations confirm that the possibility to form ternary phases at the interface between pre-made Au and Ag_2_X solid NPs is the main driving force for the formation of the hybrids. Our results point to an electrochemical replacement mechanism occurring solely through the solid interface between the two inorganic sections after their hetero-attachment.

## Experimental Section

### Chemicals

Sulfur powder (S, 99.99%), silver chloride (AgCl, 99.9%), selenium powder (Se, 99.9%), and tri-n-octylphosphine (TOP, 97%) were obtained from Strem Chemicals. Silver nitrate (AgNO_3_, 99%), gold(III) chloride trihydrate (HAuCl_4_·3H_2_O, ≥99.9%), oleylamine (OLAm, 70%), tri-n-octylphosphine oxide (TOPO, 99%), sodium borohydride (NaBH_4_, 98%), tetrahydrofuran (THF, 99%), octadecene (ODE, 90%), and toluene (99.9%), 11-mercaptoundecanoic acid (MUA, 95%), and sodium citrate tribasic dihydrate were purchased from Sigma-Aldrich. Ethanol (EtOH, 96%) and acetone (99.5%) were obtained from Panreac.

### Synthesis of Au NPs (3.5 nm)

A mixture of 20 mg (0.1 mmol) of HAuCl_4_·3H_2_O and 2 mL of ODE was degassed in three cycles of vacuum/N_2_ at room temperature followed by additions of 100 μL of TOP and 100 μL of OLAm under a N_2_ atmosphere. Subsequently, a suspension of 6 mg (0.16 mmol) of NaBH_4_ and 0.5 mL of THF was injected into the reaction mixture, and the mixture was reacted under stirring for 2 h. The solution was cleaned first by adding some milliliters of toluene to remove the excess of NaBH_4_ and centrifuged for 10 min at 4500 rpm. Then, the NPs were washed with acetone, centrifuged for 30 min at 6000 rpm, and re-dispersed in 4 mL of toluene with a concentration of 29.96 μmol/L Au NPs.

### Synthesis of Ag_2_S NPs (16 nm)

The synthesis was adapted from the work of Yang and co-workers.^27^ Briefly, 17 mg (0.1 mmol) of AgNO_3_, 8 mg of S (0.25 mmol), and 8 mL of OLAm were placed in a three-necked flask, and the mixture was purged three times by vacuum-N_2_ cycles. Afterward, the reaction temperature was raised to 160 °C under a N_2_ atmosphere. After 20 min of reaction, the heating was removed, and the solution was left to cool down to room temperature naturally. The final dark brown solution was washed once with EtOH and centrifuged for 4 min at 4500 rpm. The final NPs were re-dispersed in 4 mL of toluene for further use. The final solution is dark green with a concentration of 2.7 μmol/L Ag_2_S NPs.

### Synthesis of Ag_2_Se NPs (8 nm)

The synthesis of Ag_2_Se NPs followed the procedure published by Sahu and co-workers.^28^ Briefly, two precursor solutions were prepared first in the glove box: 474 mg (6 mmol) of Se was dissolved in 6 mL of TOP, and 572 mg (4 mmol) of AgCl was dissolved in 4 mL of TOP. Afterward, a solution of 7.8 g of TOPO and 6.6 mL of OLAm was degassed under vacuum at 120 °C for 30 min, and the temperature was then raised to 180 °C under a N_2_ atmosphere followed by the injection of Se-TOP. Once the temperature was back to 180 °C, the AgCl-TOP was injected swiftly. The heating was stopped after 20 min, and the solution was cooled down naturally. Five milliliters of toluene was added into the reaction mixture at 50 °C to prevent the solidification of the solvent. Finally, the solution was washed twice with EtOH, centrifuged for 4 min at 4500 rpm, and re-dispersed with 4 mL of toluene, resulting in a dark brown solution with a concentration of 3.6 μmol/L Ag_2_Se NPs.

### Phase Transfer of Ag_2_S and Au from Toluene to Water

Twenty milligrams (0.1 mmol) of MUA was added into a 1 mL dispersion of Ag_2_S NPs and Au NPs in toluene. Subsequently, the dispersions were brought to sonication for 15 min until the precipitates appeared at the bottom of the vials. Afterward, 1 mL of Milli-Q water was tuned to slightly basic (pH in between 7 and 8) by adding sodium citrate and added into both mixtures. Finally, the water phases for both samples were extracted and then followed with sonication for 10 min.

### Synthesis of Au–Ag_2_S and Au–Ag_2_Se Hybrid NPs

In order to obtain both hybrid systems, a simple synthetic procedure was developed. For the Au–Ag–S system, 350 μL of the Ag_2_S colloidal suspension was mixed with 50 μL of the Au suspension at room temperature for 24 h. In the case of the Au–Ag–Se system, 65 μL of the Au suspension was mixed with 350 μL of the Ag_2_Se suspension. Both reactions were stopped by adding an antisolvent of acetone followed by centrifugation for 4 min at 4500 rpm. Both final NPs were re-dispersed in toluene.

## Characterization Methods

### Transmission Electron Microscopy (TEM)

All of the samples were prepared for observation by transmission electron microscopy (TEM) by dispersion in toluene followed by sonication. A droplet was subsequently deposited on a copper TEM grid covered with holey carbon. For morphological characterization, the samples were examined in a Tecnai Spirit TEM working at 120 kV. The samples were further observed in a JEOL 2010F TEM at 200 kV and in a ThermoFisher TITAN Themis at 200 kV. The electron tomography experiments were conducted by acquiring a projection series from −70 to +70 degrees with an angular step of 5° in the same TITAN Themis at 200 kV. The X-EDS spectrum images were also acquired in the TITAN Themis at 200 kV via the Super-X in-column detector.

The optical characterization was carried out in a Cary 100 SCAN 388 Varian UV–vis spectrophotometer with quartz cuvettes. The instrument was commanded with Varian UV v. 333.

X-ray diffraction (XRD) spectra were acquired with a PANalytical X′pert Pro MPD Alpha 1 diffractometer operating in a θ/2θ geometry at 45 kV, 40 mA, and λ= 1.5406 *Å* (Cu K_α1_). Thin layers of samples were prepared by drop-casting and evaporation of the solvent in a monocrystalline Si holder of 15 mm in diameter and 0.15 mm in height. Scans in the range of 2θ = 4–100° were run at a step size of 2θ= 0.017° and 100 s per step. The data were treated with X’pert HighScorePlus software.

The composition and concentration of the NP solutions were determined by inductively coupled plasma-atomic emission spectroscopy (ICP-ES). The measurements were carried out by an Optima 3200 RL PerkinElmer spectrometer. For those measurements, 50 μL of solutions was precipitated in MeOH and redispersed in CHCl_3_. The solution was evaporated in an oven overnight at 90 °C. Before the vial was sealed, 2.5 mL of aqua regia was added to the precipitate and then heated to 90 °C for 72 h. The resulting solution was transferred to a 25 mL volumetric flask and diluted with Mili-Q water.

Zeta potentials for the above-described Ag_2_S and Au (3.5 nm) NPs were monitored using a ZetasizerNano ZS (Malvern Instruments Ltd., Germany).

### Computational Section

To gain insight into the relative stability of the observed phases and to determine their electronic band gap, density functional theory (DFT) calculations were conducted using the Vienna ab initio simulation package (VASP).^29,30^ The projector augmented wave (PAW) method^31,32^ was applied in combination with the generalized gradient approximation (GGA) by Perdew, Burke, and Ernzerhof (PBE).^33^ Settings for the energy cutoff of the electronic wavefunctions and for the density of the k-mesh were tested on elemental Ag, elemental Au, and orthorhombic Ag_2_Se to ensure energy convergence within 0.5 meV/atom. Settings meeting this criterion were subsequently applied to all phases. Consequently, all structures were calculated using energy cutoffs of 550 eV for the valence electronic wavefunctions and 770 eV for the augmentation wavefunctions. For the metallic systems of Ag, Au, and Ag_*x*_Au_*y*_, the required k-mesh was 28 × 28 × 28 for the conventional fcc unit cells. For all compounds of M_2_S and M_2_Se (M = Ag, Au), the k-meshes were set to have a linear k-spacing of less than 0.028 Å^–1^ in any reciprocal lattice direction. Energy convergence criteria of 10^–6^ and 10^–5^ eV were used for the electronic and ionic loops, respectively. Table S3 provides an overview of all calculated phases with the number of atoms per unit cell and the k-meshes used in the calculations. During the GGA-PBE calculations, both the cell dimensions and the atomic coordinates were fully relaxed to obtain the lowest-energy configurations. The calculated lattice parameters are also provided in Table S3.

The DFT calculations are valid for a temperature of 0 K and a pressure of 0 Pa. Spin–orbit coupling effects were not taken into account. For calculation of Ag–Au mixed phases, ordered Ag_3_Au_1_ and Ag_1_Au_1_ compounds were calculated within the conventional fcc unit cell. The entropy of mixing in the Ag–Au system, which is known to form a continuous solid solution, has therefore been ignored as well. For the AgAuS *petrovskaite* phase, there is some discussion in the literature about the exact composition as the 9e Wyckoff position is reported to be 1/3-occupied by Ag atoms and 2/3-occupied by Au atoms.^34^ To cover a wider compositional range, configurations with all 9e Wyckoff positions occupied by either Ag or Au atoms were also calculated within the *petrovskaite* phase (yielding compositions of Ag_30_Au_18_S_24_ and Ag_21_Au_17_S_24_), but these were found to be energetically relatively unfavorable.

As DFT calculations typically lead to severe underestimation of the size of the electronic band gap, hybrid DFT calculations were also performed using the Heyd–Scuseria–Ernzerhof HSE06 functional^35^ with 25% of Hartree-Fock exchange. The HSE06 calculations were performed on the relaxed configurations as found from GGA-PBE without any further relaxation.

## Results and Discussion

### Ag_2_S Precursor NPs and Au–Ag_2_S Hybrid NPs

**Figure 1:**
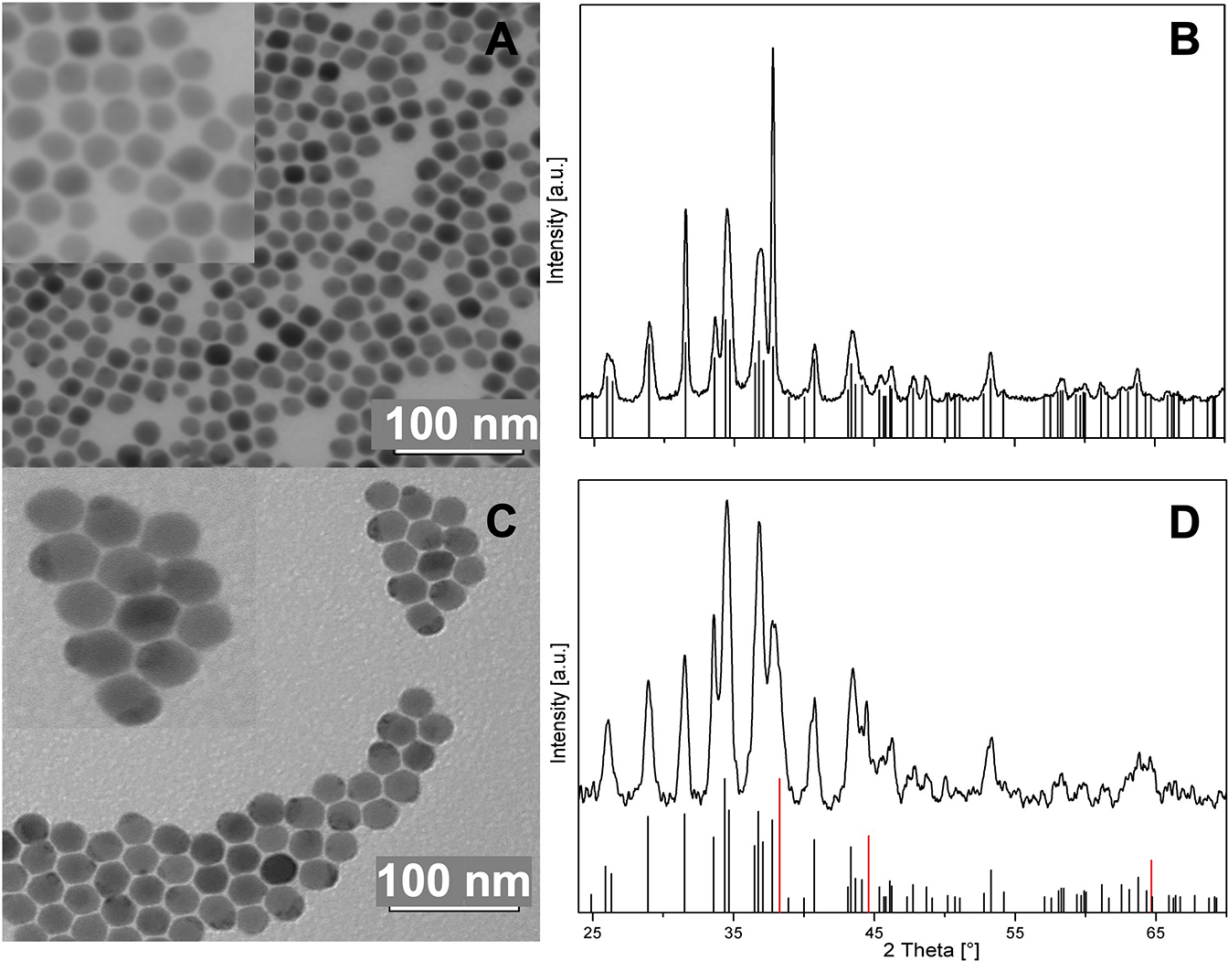
(A) TEM micrograph of Ag_2_S NPs. (B) XRD spectrum of the Ag_2_S NPs and Ag_2_S (JCPDS no. 00-024-0715, black) reference pattern. (C) TEM images of Au–Ag_2_S hybrid NPs. (D) XRD spectrum of the Au–Ag_2_S hybrid NPs, Ag_2_S (JCPDS no. 00-024-0715, black), and Au (JCPDS no. 00-001-1172, red) reference pattern.

For the synthesis of the Au–Ag_2_S hybrid system, Ag_2_S NPs and Au NPs were used as precursors for further mixing. The Ag_2_S precursors were prepared by a heat-up method published by Wang and co-workers,^27^ which is a one-pot reaction consisting of AgNO_3_, S, and oleylamine as both a surfactant and solvent heated up to 160 °C under an inert atmosphere, resulting in quite homogeneous faceted NPs with a diameter of 16 nm, as shown in the TEM micrograph in Figure 1A. The XRD spectrum in Figure 1B showed the formation of Ag_2_S, crystallizing in an acanthite monoclinic structure as expected, that is stable below a temperature of 177 °C.^36^ A small fraction of NPs contained an additional smaller and darker domain at their surface, as observed by low-resolution TEM micrographs. They were further analyzed by high-resolution TEM (HRTEM), as shown in Figure S1, and they were identified as metallic Ag appearing on the surface of a few monoclinic Ag_2_S NPs.

The formation of Au–Ag_2_S heterodimers was achieved by simply mixing the two pre-synthesized Ag_2_S NPs and Au NPs in solution at room temperature. A TEM micrograph of the precursor Au NPs is shown in Figure S2: they exhibit a spherical shape with an average diameter of 3.5 nm. The TEM micrograph in Figure 1C shows the nanostructures obtained upon mixture of the NPs. A clear second domain with a dark contrast is observed on almost each surface of the faceted Ag_2_S NPs. The XRD, shown in Figure 1D, reveals the presence of cubic metallic Au in the samples through the extra “shoulder” peak at 38.2° and another peak at 44.6° that are the two most intense peaks belonging to cubic metallic Au.

**Figure 2:**
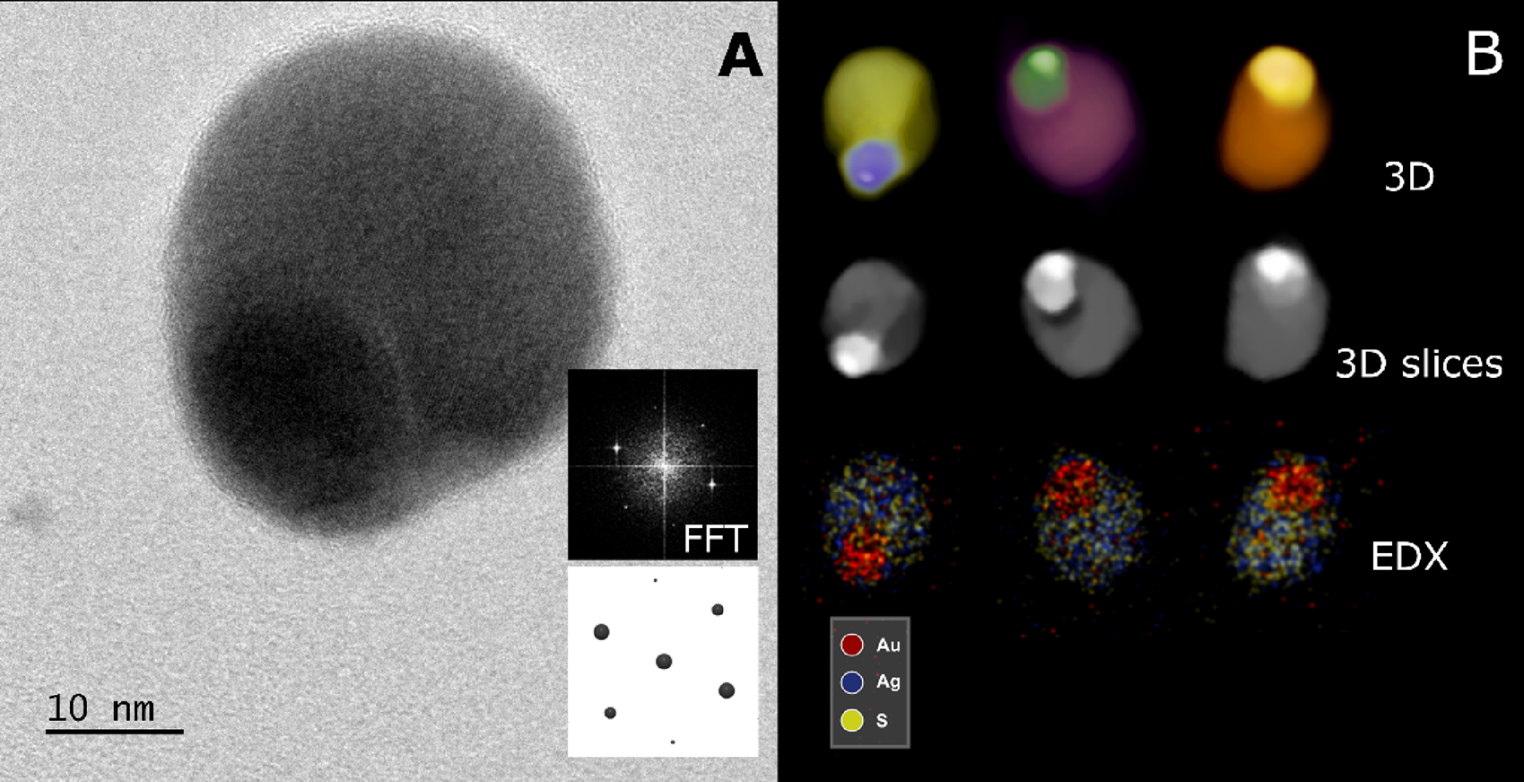
Au–Ag_2_S hybrid NP characterization. (A) HRTEM image with FFT as an inset. The crystal could be indexed as a [352]-oriented acanthite (see theoretical pattern below the FFT). (B) Electron tomographic reconstructions of three particles (shown as volume renderings in arbitrary colors) with an orthoslice through the volume of each particle and elemental mappings extracted from X-EDS spectra.

The HRTEM image and the result of STEM-EDS elemental maps in Figure 2A,B reflect the same results derived previously by XRD analysis. The volume renderings in Figure 2B obtained through electron tomography only show the presence of two domains in every particle. The two intensities observed in the images correspond to Ag_2_S for the lowest intensity and Au for the highest. The slices through the reconstructed volume show how the Au part is partially physically embedded in the Ag_2_S, and the EDX maps show the confinement of Au exclusively to the bright domain. The HRTEM image in Figure 2A shows atomic planes that could be indexed as acanthite Ag_2_S, thus confirming the nature of the largest domain.

### Ag_2_Se Precursor NPs and Au–Ag_2_Se Hybrid NPs

**Figure 3:**
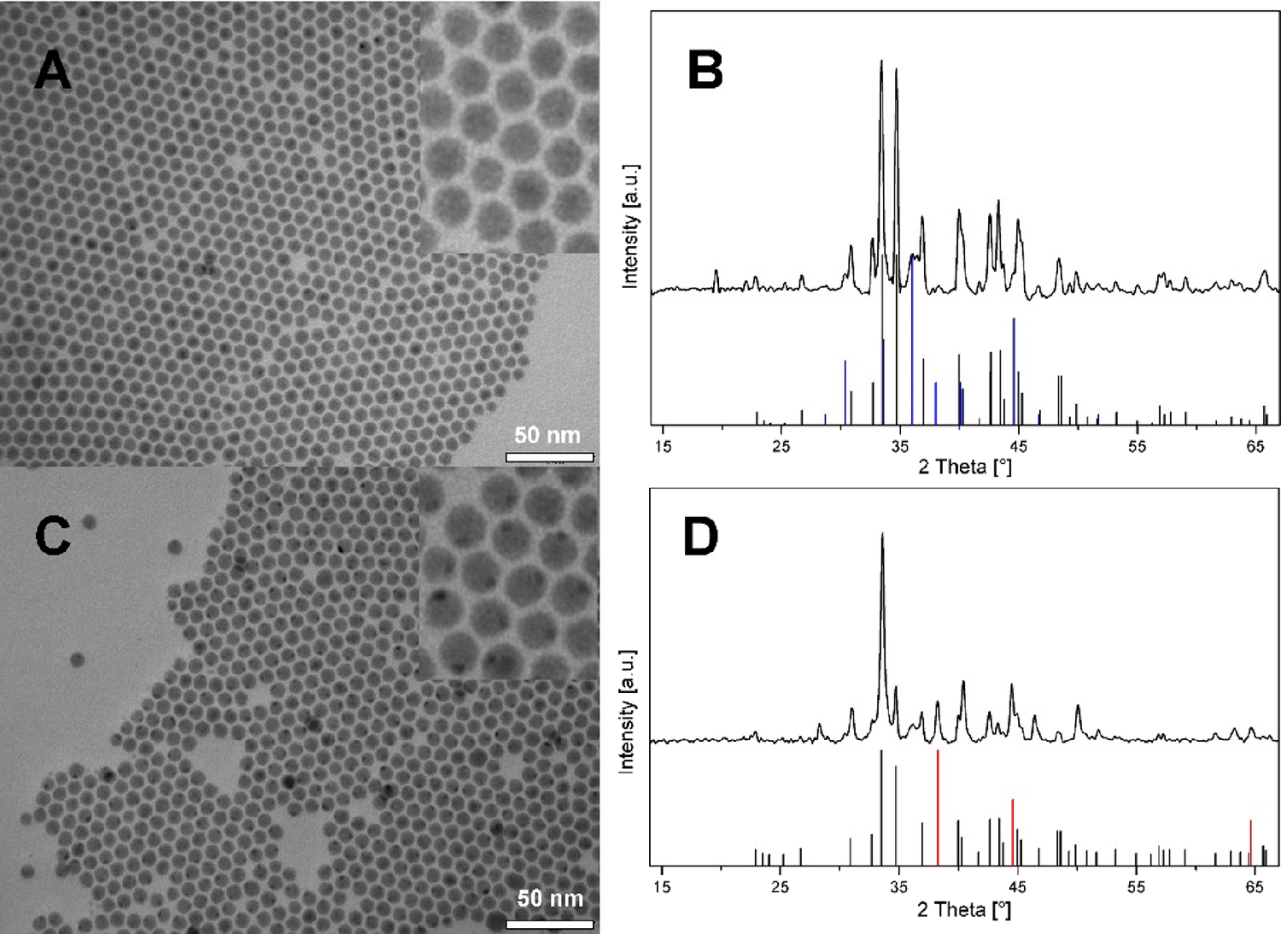
(A) TEM micrograph of Ag_2_Se NPs. (B) XRD spectrum of the Ag_2_Se NPs, β-Ag_2_Se (JCPDS no. 00-024-1041, black) reference pattern, and t-Ag_2_Se calculated (blue) pattern.^28^ (C) TEM images of Au–Ag_2_Se hybrid NPs. (D) XRD spectrum of the Au–Ag_2_Se hybrid NPs, Ag_2_Se (JCPDS no. 00-024-1041, black), and Au (no. JCPDS 00-001-1172, red) reference pattern.

The synthetic approach begins first with the preparation of monodisperse Ag_2_Se NPs as precursors by a hot-injection method developed by Yang and co-workers.^37^Figure 3A shows the TEM micrograph of as-synthesized Ag_2_Se NPs with a hexagonal shape and with an average diameter of 8 nm. The XRD pattern of the sample in Figure 3B indicates that the NPs crystallized mainly in the orthorhombic phase (β-Ag_2_Se), which is one of the three known crystallographic phases for Ag_2_Se. The others are in the cubic phase (α-Ag_2_Se), which is stable above 135 °C, and metastable tetragonal phase (τ-Ag_2_Se), exclusively observed in nanocrystals or polycrystalline Ag_2_Se contained in thin films.^38^ The latter phase can also be identified in the XRD spectrum as a minor product. The formation of Au–Ag_2_Se NPs is performed analogously by the same strategy used for the preparation of hybrid Au–Ag_2_S NPs in the previous section. The final product was observed by TEM and a general view is shown in Figure 3C. In comparison with the TEM images of Ag_2_Se precursors, a small dark spot appears attached on the surface of almost every hexagonally shaped Ag_2_Se NP, corresponding to metallic Au domains (as observed in Figure S3), indicating the formation of hybrid Au–Ag_2_Se systems. The corresponding XRD spectrum in Figure 3D confirms our assumption, showing that some additional peaks arise besides those belonging to orthorhombic Ag_2_Se, which can be assigned to cubic metallic Au: see its most intense and characteristic diffraction peak at 38.2°, which is attributed to the Au(111) set of equivalent planes.

### Au–Ag_3_AuS_2_–Ag_2_S NPs and Au–Ag_3_AuSe_2_–Ag_2_Se NPs

**Figure 4:**
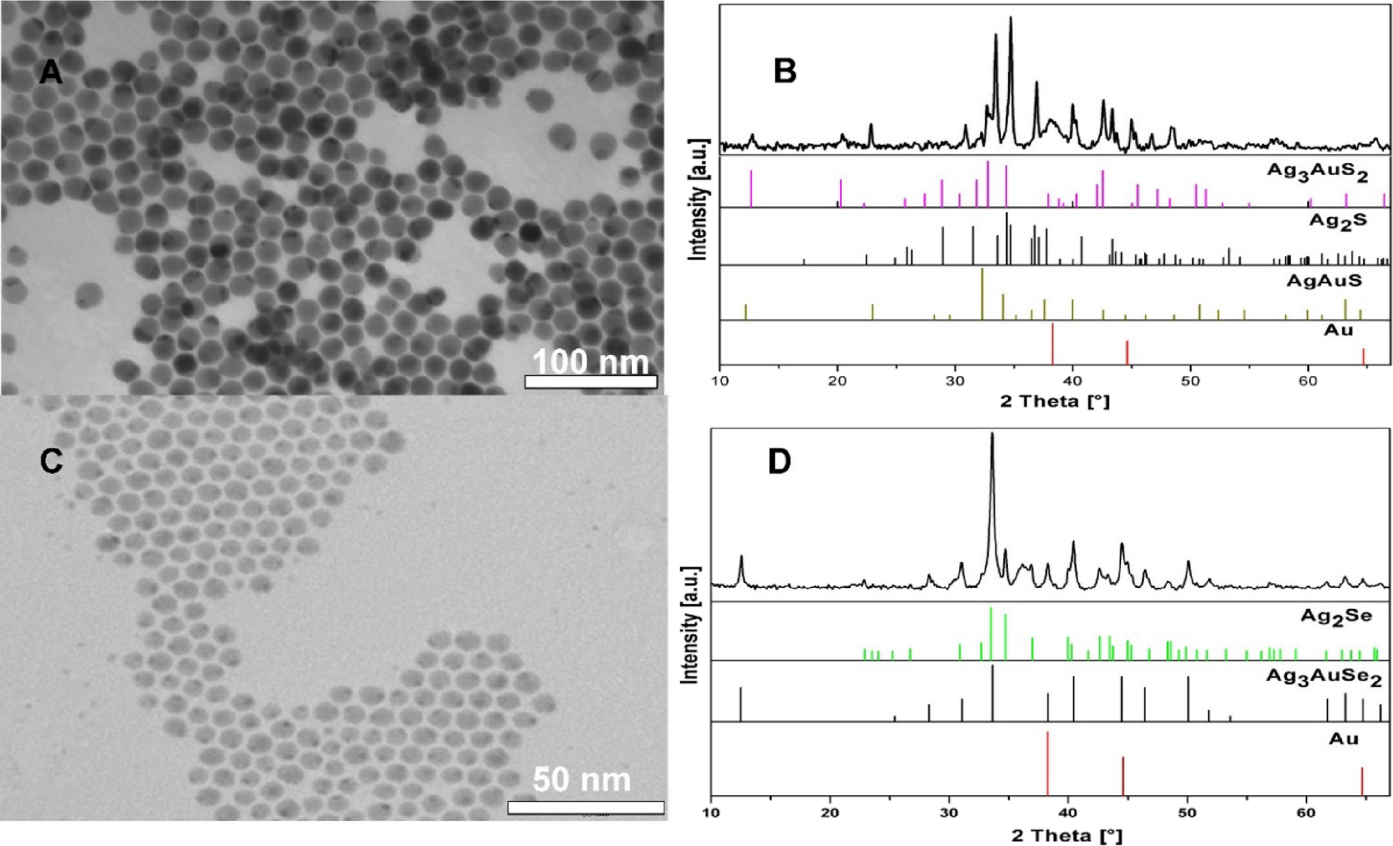
(A) TEM micrograph of the Au–AuAg_3_S_2_–Ag_2_S sample. (B) XRD spectrum of Au–Ag_3_AuS_2_–Ag_2_S, Ag_2_S (JCPDS no. 00-024-0715, black), AgAuS (JCPDS no. 00-038-0396, olive), Ag_3_AuS_2_ (JCPDS no. 01-072-0390, pink), and Au (JCPDS no. 00-001-1172, red) reference pattern. (C) TEM images of the Au–Ag_3_AuSe_2_–Ag_2_Se sample. (D) XRD spectrum of Au–AuAg_3_Se_2_–Ag_2_Se, Ag_2_Se (JCPDS no. 00-024-1041, black), Ag_3_AuSe_2_ (JCPDS no. 00-025-0367, green), and Au (JCPDS no. 00-001-1172, red) reference pattern.

**Figure 5:**
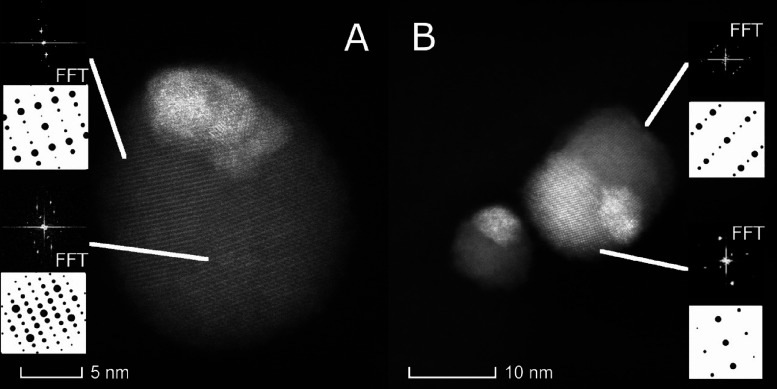
HRSTEM images of (A) Au–Ag_3_AuS_2_–Ag_2_S NPs and (B) Au–Ag_3_AuSe_2_–Ag_2_Se NPs. The FFTs of the two biggest crystallites observed in the particles have been indexed as [101] Ag_3_AuS_2_ (top inset) and [101] Ag_2_S (bottom inset) for A and [212] Ag_3_AuSe_2_ (top inset) and [241] Ag_2_Se (bottom inset) for B. The brightest crystal corresponds to gold.

The complexity of crystalline phases in those two Au–Ag–S and Au–Ag–Se systems can be further tuned by doubling the amounts of Au NPs in both reactions. Although the TEM micrographs at low magnification of both samples (Figure 4A,C) are very similar to those of the previous hybrid NPs (Au–Ag_2_S and Au–Ag_2_Se) in morphology and size, their corresponding XRD spectra (Figure 4B,D) show that both samples are not composed of only the binary precursors and metallic Au. The extra peaks of the Au–Ag–S sample in the XRD spectrum (Figure 4B) show the formation of the cubic Ag_3_AuS_2_ phase besides the acanthite Ag_2_S. Moreover, a monoclinic lattice from another AgAuS ternary material is also very likely contained in the sample mainly through two assignable peaks at 22.9° and 39.9°, although this cannot be fully confirmed due to the fact that the reference pattern of AgAuS always overlaps with one or two other reference patterns (Ag_3_AuS_2_ and Ag_2_S). By analyzing the XRD pattern of the selenium-containing sample (Au–Ag–Se), a few relative intense peaks located at 41.3°, 28.3°, and especially 12.5°, representing quite a large interplanar distance, can be easily assigned to the (310), (420), and (111) sets of planes in the cubic Ag_3_AuSe_2_ crystal structure. In Figure 5, one HR-HAADF image per material is shown with three crystalline regions each. As observed previously, the smallest and brightest region corresponds to metallic Au. Upon close examination, the other crystallites can be indexed as the binary and ternary phases of Au–Ag_3_AuS_2_–Ag_2_S (A) and Au–Ag_3_AuSe_2_–Ag_2_Se (B), which corroborates and refines the XRD observations. In both kinds of particles, the ternary and binary compounds are in contact with the gold domain, but the ternary ones share a vaster interface with it.

### Study of Influencing Factors and the Reaction Mechanism

Analogous reactions of Ag_2_S NPs have been performed with Au NPs of different sizes, with Au nanorods (NRs) and with hydrophilic Ag_2_S and Au NPs in order to unveil the size, shape, ligand, and solvent dependence of the reaction studied. The use of 11 nm Au NPs as precursors instead of 3.5 nm ones did not present any change in the final product obtained besides the obvious fact of obtaining Au–Ag_2_S dimers with larger Au domains, as shown by TEM and XRD in Table S1. However, using 25 nm Au NPs as precursors led to an irreversible aggregation of Au after long reaction times, and no hybrid Au–Ag_2_S NPs could be observed, as indicated in Table S1 and Figure S4, through TEM, XRD, and EDX analysis. The use of anisotropically shaped Ag_2_S NRs instead of spherical NPs neither presented any inconvenience for the occurrence of the reaction as shown in Figure S5. Lastly, ligand exchange reactions were performed in both nanostructured precursors, resulting in water-dispersed 3.5 nm Au NPs and 16 nm Ag_2_S NPs, both capped with MUA instead of OLAm. Subsequently, both NPs were mixed under the same reaction conditions as in the initially reported synthesis but in water. As a result, analogous dimer-like NPs were formed, as shown in Table S1. All in all, these additional experiments suggest that the current method is not restricted to a certain type of ligand, solvent, and shape of pre-made NPs. However, the methodology seems to be applicable only for small or medium-sized Au NPs (up to 11 nm based on the experiments) since relatively large Au NPs tend to aggregate with each other rather than react with Ag_2_S NPs. It is also interesting to note that the formation of ternary phases (Ag_3_AuS_2_, AgAuS, or Ag_3_AuSe_2_) has been confirmed in all samples prepared both by bulk XRD and local HRTEM analysis, as shown in Table S1 and Figure S6.

The heterodimers found in these samples are very similar to those obtained through standard seeded-growth methods, as reported in our previous work, in terms of size, geometry, and homogeneity.^9,26^ Nevertheless, this new procedure requires no phase transfer of Au(III) ions from water to toluene since pre-made Au NPs are used as precursors. Additionally, the heterogeneous nucleation of Au domains on the surface of chalcogenide NPs occurring in seeded-growth approaches requires the presence of surfactant-stabilizing molecules in considerable amounts, which might not only present an issue in terms of contamination for their characterization, but more importantly, their electronic and catalytic performance can fade.

**Figure 6:**
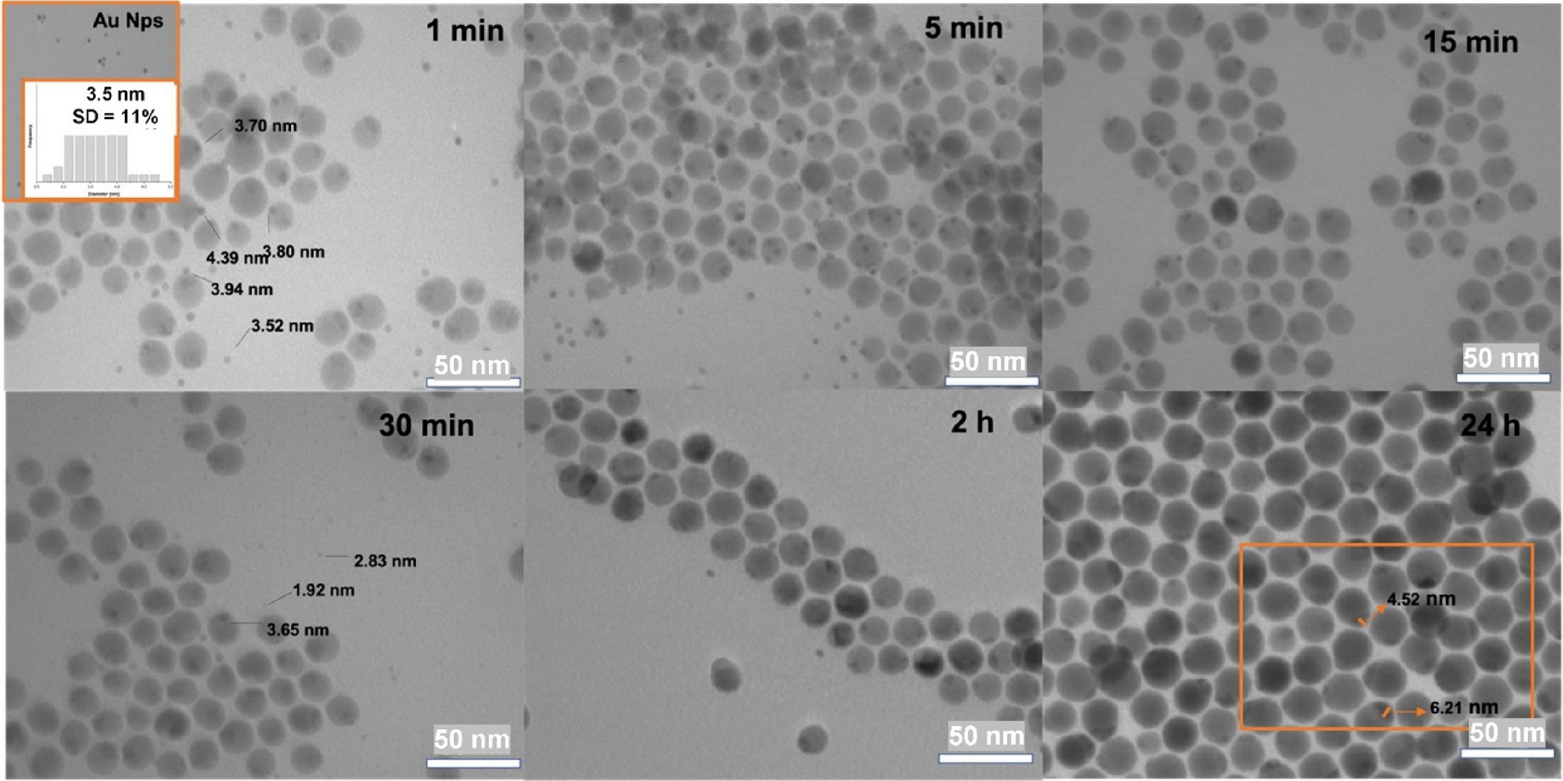
TEM images of aliquots at specific times along the reaction between Ag_2_S NPs and Au NPs.

**Scheme 1:**
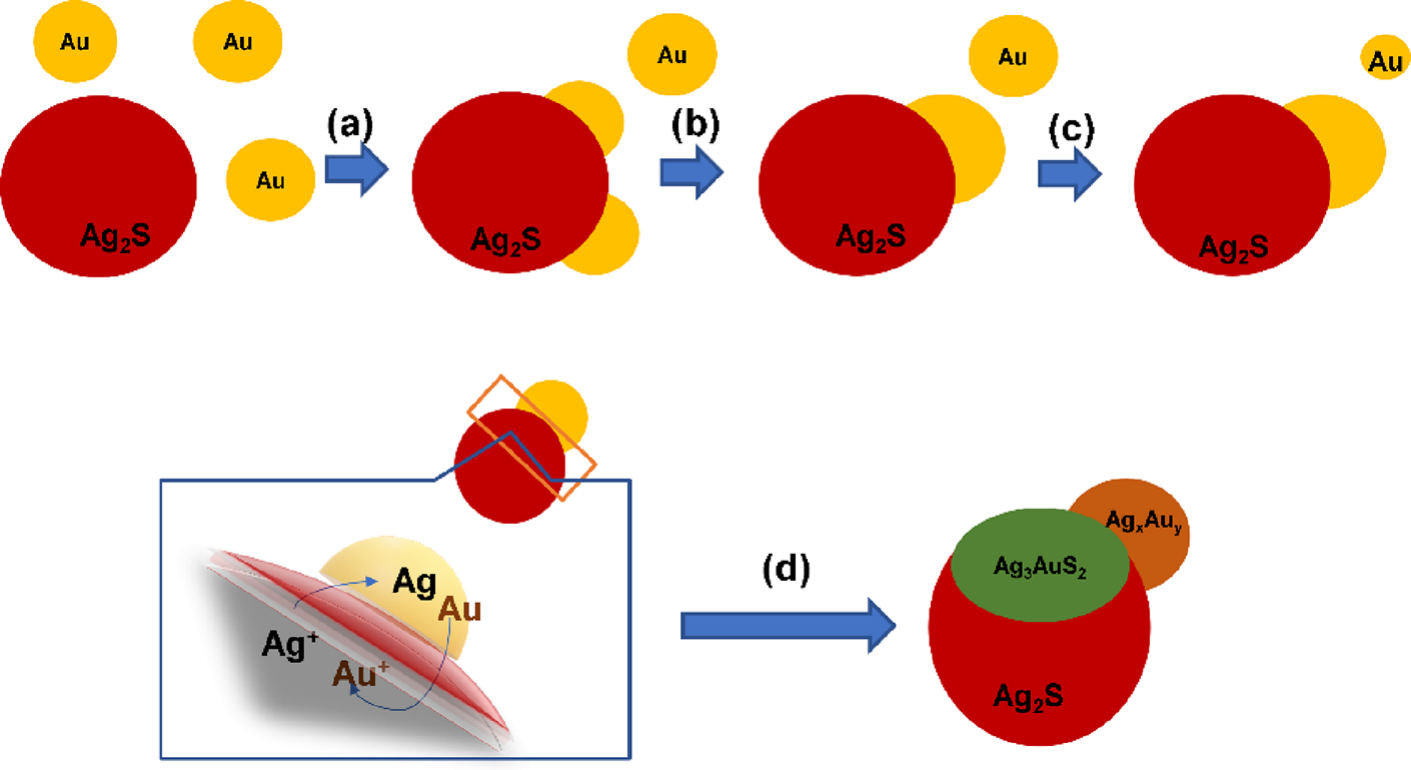
Heterodimer Formation Mechanism (a) NP hetero-attachment, (b) Au NP coalescence at the chalcogenide surface, (c) solution-mediated Au NP ripening, and (d) solid interface-confined electrochemical replacement.

The reaction mechanism has been investigated through an experiment in which Ag_2_S NPs and Au NPs were mixed following the method described in the Experimental Section, and aliquots were withdrawn from the reaction flask at specific time lapses and analyzed under the TEM. Figure 6 shows how the aliquot taken at 1 min contains single-component Ag_2_S and Au NPs as major products, although a considerable amount of Au–Ag_2_S hybrid NPs can be already identified after a short reaction time. In the course of the reaction, the population of hybrid nanostructures becomes significantly larger until every Ag_2_S NP is decorated with at least one Au domain, while isolated Au NPs are still observed in the reaction mixture due to the excess of Au NPs added to the reaction medium. Noteworthy, size analysis indicates that Au domains preserve the size of the original precursor NPs for up to ca. 15 min of reaction, both the ones already attached to the semiconductor domain and those still free in the solution. These observations might be seen as an evidence of direct particle hetero-attachment as the single operative mechanism during the formation of solid–solid Au–Ag_2_X interfaces, thus confirming the absence of ripening of Au NPs and their heterogeneous nucleation at the surface of Ag_2_S. The Au/Ag_2_X NP ratio in the reaction medium plays an important role in tuning the size of the final Au domain, as inferred from the Au–Ag_2_S NPs with large Au domains seen in Figure 1C where a Au/Ag_2_S NP ratio larger than 1 was used, compared to Au–Ag_2_Se NPs with smaller Au domains in Figure 3C where the Au/Ag_2_Se NP ratio was closer to unity. Indeed, Au–Ag_2_S hybrid NPs prepared with a Au/Ag_2_S NP ratio close to 2 show many particles with two or even three Au NPs attached during the first 15 min of reaction. Interestingly, for longer reaction times, these multi-domain NPs evolve to dimer-like NPs where the Au domain grows and significantly modifies its initial shape. Simultaneously, the Au NPs that are still free in solution are observed to gradually decrease their size, as confirmed by size analysis. All in all, our experiments point to direct particle hetero-attachment as the single operative mechanism during the formation of solid–solid Au-Ag_2_X interfaces at short reaction times, although in those systems where Au/Ag_2_X NP ratios larger than 1 are used, the final size of the Au domain in the hybrid NP can be increased through (a) the coalescence of multiple Au NPs already attached at the chalcogenide surface and (b) a solution-mediated ripening process involving free Au NPs in solution used as sacrificial dots, as illustrated in Scheme 1. Based on the data shown in Figure S7, identical conclusions can be extracted from those experiments performed using larger Au NPs as precursors (ca. 11 nm), suggesting that the reaction mechanism is valid within the full range of sizes for which the reaction occurs, as stated above.

**Figure 7:**
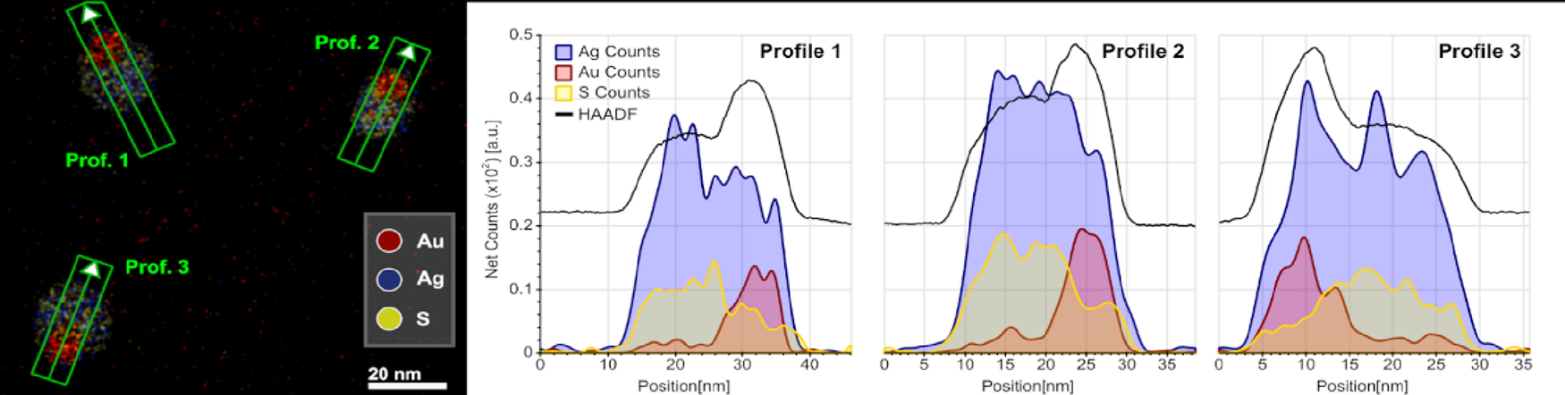
EDX map of three Ag_3_AuS_2_ particles from which signal profiles have been extracted along the indicated arrows. Note the presence of the Ag signal all over the particle.

In those experiments with a Au/Ag_2_X ratio of above 3, the formation of ternary Ag_3_AuX_2_ phases is always confirmed after relatively long reaction times. Indeed, ternary domains are strongly confined at the newly formed solid–solid interface between the metallic and the semiconductor sections, suggesting that their formation is strictly related to an interface phenomenon. Additional measurements (Figure 7) indicate the presence of Ag within the metallic Au domains. The EDX elemental profiles extracted along the particles show the presence of Ag all over the analyzed particles. Although not quantifiable, the data confirm the partial reduction of Ag^+^ cations from the Ag_2_X domain to metallic Ag, which are alloyed with the Au domain. Clearly, the partial reduction and release of Ag^+^ cations from the Ag_2_X domain is accompanied by the partial oxidation of metallic Au atoms to Au^+^ cations that diffuse into the semiconductor to form the observed Ag_3_AuX_2_ ternary interface, as depicted in Scheme 1 and indicated in the following reaction for the Se-based system:
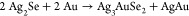
1

Thus, it can be concluded that the stabilization of the interface takes place through an interdomain redox reaction at the solid–solid interface followed by atomic diffusion and exchange, occurring only after particle hetero-attachment, leading to the formation of ternary semiconductor phases and metallic alloyed domains. Compared to previous strategies^9,26^ where more standard molecular precursors are used for the exchange leading to ternary phases (i.e., AuCl_3_ instead of Au NPs), the replacement here described occurs exclusively at the interface and can be considered as an intraparticle process: ternary domains grown far apart from the metal–semiconductor interface were never found, suggesting that the solvent is not assisting the replacement through atom diffusion, which is only a solid-phase phenomenon.

### Stability of the Ternary Phases

Both Au and Ag_2_X precursor NPs are stabilized in toluene by coordinating OLAm, a long-chain hydrocarbon amine. This fact excludes the electrostatic attraction between oppositely charged NPs as a driving force promoting hetero-attachment (asymmetric Au–Ag_2_X) versus homo-attachment (symmetric Au–Au or Ag_2_X–Ag_2_X) of NPs in solution in contrast with previous reports.^39,40^ For further evidence, zeta-potential measurements were performed for Au–MUA and Ag_2_S–MUA hydrophilic NPs in water, which suffer analogous hetero-attachment upon mixture. Negative zeta-potential values were obtained in both cases, as shown in Table S2. These data indicate that the hetero-attachment occurs regardless of the electrostatic repulsion between Au NPs and Ag_2_X NPs in water.

Thus, one may think of surface tension reduction as the main factor facilitating the attachment. However, if this was the only factor to consider, homo-attachment is expected to reduce even more the surface energy in the system compared to hetero-attachment due to the optimal lattice match and chemical affinity in the first case. In all our experiments, no dimer particles made of a single component were ever observed, indicating that the precursor NPs are well isolated and stabilized as colloids by the surfactants at their surface, and no attachment or aggregation is needed to decrease the energy of the system. Moreover, the hetero-attachment observed is not crystallographically oriented, that is, no epitaxial relationship could be found at the interface between the two domains. This is a further indication that there is no specific combination of crystal facets that manages to stabilize a net Au–Ag_2_X interface and would be directing the attachment. Consequently, there must be another driving force that leads exclusively to the hetero-attachment observed, which is most likely the ease of the formation of the thermodynamically stable ternary compounds.

The experimental data collected confirm that metallic Au is oxidized to Au^+^ while Ag^+^ reduces to metallic Ag to form the ternary chalcogenide and the alloy at the interface, respectively. This proceeds spontaneously, although the standard reduction potentials of those metals would dictate the opposite reaction.^41^ Recently, Pattadar et al. reported on the observation of the so-called size-dependent antigalvanic replacement between Au NPs and Ag^+^ solution where the unfavorable thermodynamic redox process was gaining importance with the decreasing size of the Au NPs.^42^ In view of our results, a similar redox replacement is observed, although in the solid state. Additionally, when using differently sized Au NPs (see previous section), it can be concluded that the solid-state redox replacement observed is also size-dependent. Indeed, the solid state electrochemical replacement is especially favored in the case of small (<11 nm) and less stable Au NPs, becoming in these cases a viable mechanism for the formation of the thermodynamically stable ternary phase observed. However, in the case of larger and more stable Au NPs (25 nm), the system prefers to increase its thermodynamic stability through the homo-attachment of Au NPs (aggregation) rather than by hetero-attachment and the subsequent formation of a stable ternary phase. A similar unexpected solid-state replacement was observed by some of us during the transformation of Au–CdS nanostructures into Cd–Au_2_S hybrid NPs.^43^ However, in that case, the process took place only under the effect of the electron beam of the TEM, evidencing the high activation energy associated to the transformation. In contrast, the electrochemical replacement described in this work occurs spontaneously at room temperature with no specific energy input, making it kinetically feasible at room temperature.

### Atomistic First Principles Calculations

**Figure 8:**
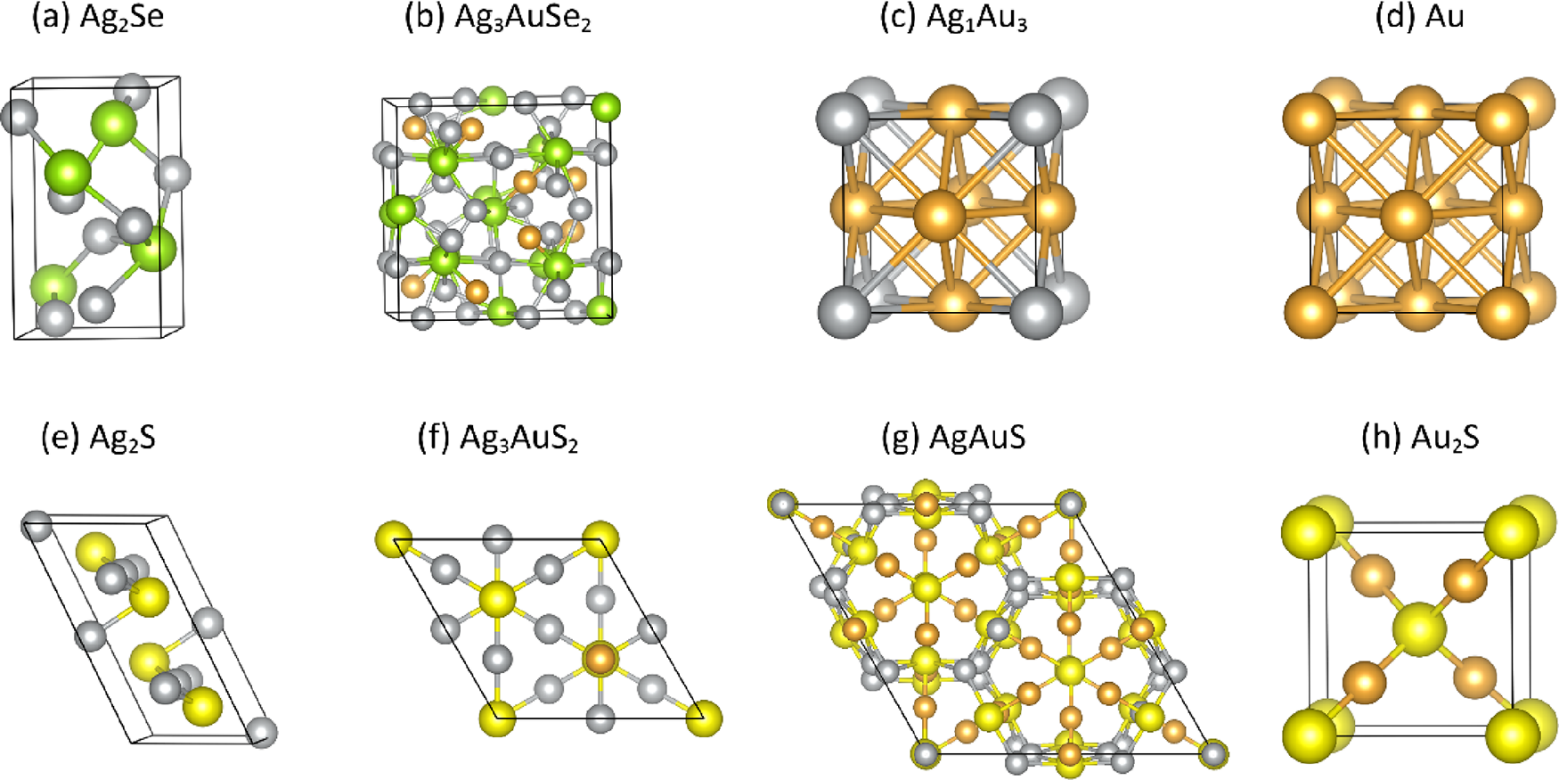
Structural models (Ag, Au)_2_Se and (Ag, Au)_2_S compounds as well as Au and Ag_1_Au_3_ metallic phases. Gold, silver, green, and yellow spheres depict Au, Ag, Se, and S atoms, respectively. The boundaries of the unit cells are indicated with solid black lines.

Density functional theory (DFT) calculations were carried out to investigate the relative thermodynamic stability of the phases involved in the process. The calculations were performed using the VASP code^29,30^ using the PBE functional^33^ for total energy calculations of the fully relaxed structures and using the HSE06 functional^35^ for the calculations of the band gaps of ternary phases, as detailed in the Computational Section. An overview of the phases considered in the calculations is provided in Table S3, and the unit cells of the considered phases as obtained after full relaxation are shown in Figure 8.

From the calculated total energies, the relative thermodynamic stability of the ternary phases (*fischesserite*, *uytenbogaardite*, and *petrovskaite*) was evaluated, starting with *fischesserite* Ag_3_AuSe_2_.

Assuming that the *fischesserite* phase is formed from Ag_2_Se and Au, the energy gain associated with the formation of this phase can be evaluated from the reaction formulas listed in Table 1. With these reactions, various possibilities are considered for the Ag atoms that are expelled from the Ag_2_Se compound and replaced with Au where pure Ag, AgAu, and AgAu_3_ may be formed. The energies at the right-hand side of Table 1 show that the formation of pure Ag and AgAu as products would be favorable but the formation of an AgAu_3_ alloy phase is most favorable. This agrees well with the experimental EDS observations showing that the metallic Au nanodomain also contains a considerable fraction of Ag.

**Table 1:** Change in the Potential Energy associated with the Formation of *Fischesserite* Ag_3_AuSe_2_ from Ag_2_Se and Au

reaction formula	Δ*E* (eV)
2(Ag_2_Se) + Au → Ag_3_AuSe_2_ + Ag	–0.230
2(Ag_2_Se) + 2Au → Ag_3_AuSe_2_ + AgAu	–0.242
2(Ag_2_Se) + 4Au → Ag_3_AuSe_2_ + AgAu_3_	–0.302

Second, the relative stability of the ternary sulfur compounds *uytenbogaardite* and *petrovskaite* was considered. Which phases are thermodynamically stable in the Ag–Au–S system depends on the relative concentrations of Ag, Au, and S. As *uytenbogaardite* and *petrovskaite* both have a cation/anion ratio of 2, to determine their thermodynamic stability, we have considered a subset of the Ag–Au–S system that can be described with the composition (Ag_1–*x*_Au_*x*_)_2_S. Within this subset, the compositional extremes for *x* = 0 and *x* = 1 correspond to the well-known Ag_2_S and Au_2_S phases, and the formation energies of the *uytenbogaardite* and *petrovskaite* phases can be defined with respect to the energies of the Ag_2_S and Au_2_S phases:
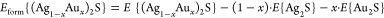
2

The formation energies thus obtained are listed in Table S4 and plotted in Figure S8. The energy of the *petrovskaite* phase is above the common tangent line, implying that the phase is not stable with respect to decomposition into *uytenbogaardite* and Au_2_S. The energy differences are very small, however, that is, less than 10 meV/atom, indicating that all these phases are relatively stable at the respective compositions (Ag/Au ratios). It is also clear from Table S4 and Figure S8 that the compositional variations of the *petrovskaite* phase that were considered are energetically unfavorable in comparison with the standard composition AgAuS of this phase.

The DFT total energy calculations show that the formation of the three ternary phases is either favorable (*fischesserite* from Ag_2_Se and Au) or likely to occur (*uytenbogaardite* and *pertrovskaite* from Ag_2_S and Au). We mention here that only bulk unit cells have been considered in the DFT calculations. At the nanoscale, surface and interface energies are of importance as well and can very well stabilize a particular phase that is expected to be less stable when considering only bulk formation energies.

All in all, the experimental data, together with the results obtained by theoretical calculations, suggest that the formation of a kinetically favored and thermodynamically stable ternary phase (Ag_3_AuX_2_) is the main driving force promoting the formation of hybrid NPs.

To explore the functional potential of the ternary phases for optoelectronic applications, we also calculated the electronic band gap of these phases using the more advanced HSE06 functional (see the Characterization Methods section). From the values of the band gaps, as listed in Table 2, it becomes clear that *fischesserite* is predicted to be a narrow-band-gap semiconductor, while *uytenbogaardite* and *petrovskaite* have band gaps corresponding to blue and red wavelengths in the visible spectrum.

**Table 2:** Electronic Band Gaps Calculated Using HSE06

compound	phase	band gap (eV)
Ag_3_AuSe_2_	*fischesserite*	0.66
Ag_3_AuS_2_	*uytenbogaardite*	2.49
AgAuS	*petrovskaite*	1.61

**Figure 9:**
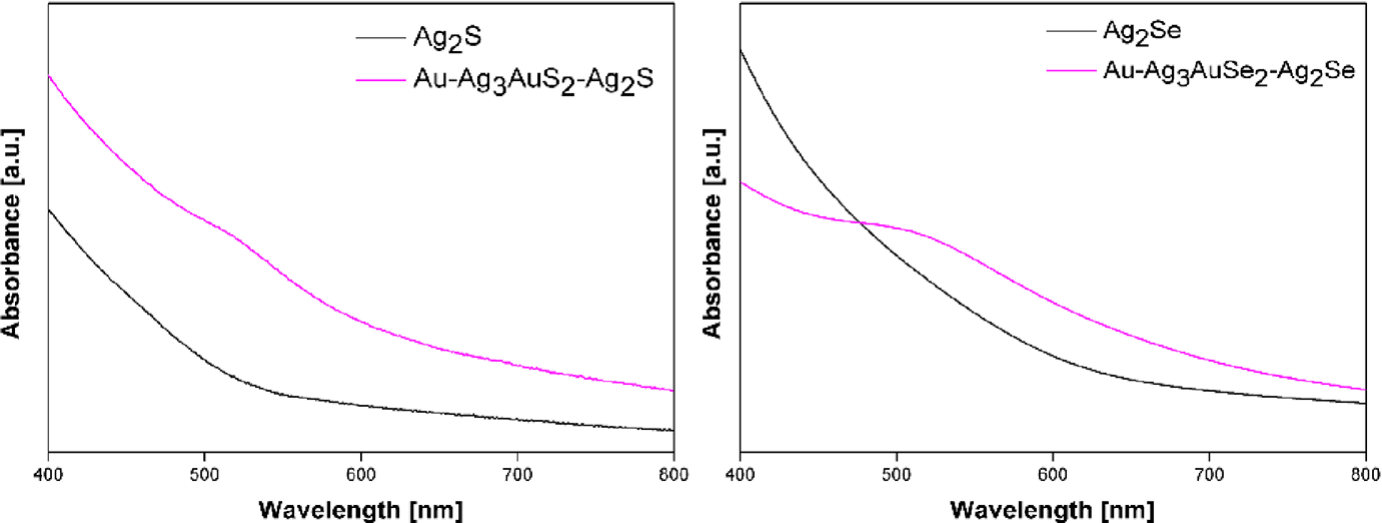
UV–vis absorption spectra of the Au–Ag_3_S_2_–Ag_2_S sample compared to Ag_2_S NPs (left) and the Au–Ag_3_AuSe_2_–Ag_2_S sample compared to Ag_2_Se NPs (right).

The measurements for optical absorption of both samples were carried out in solution in the visible range. As shown in Figure 9, both samples show a gradually increasing absorption profile and a broad absorption band centered between 500 and 600 nm, which is characteristic of the localized surface plasmon resonance of colloidal Au.

## Conclusions

In conclusion, the hetero-attachment of monometallic NPs and binary chalcogenide nanocrystals at room temperature and in solution is described, which leads to the formation of Au–Ag_2_X hybrid NPs, where X = S, Se. The high thermodynamic stability and kinetic easiness of the formation of ternary phases and alloys at the nanoscale seem to be responsible for the high yield and selectivity of the hetero-attachment with respect to the homocoalescence of NPs. Indeed, upon the formation of the solid–solid interface between the two precursors Au and Ag_2_X NPs, the growth of Au_3_Ag and Ag_3_AuX_2_ domains occurs through solid-phase diffusion and electrochemical replacement through the interface, regardless of the standard redox potentials involved. The extension of the methodology to other technologically relevant materials is expected to enhance the consolidation of interfaces in solid nanostructured composites and thin films, improving in this way the transport properties in optoelectronic, thermoelectric, and catalytic devices.

## Supporting Information Available

Synthesis of 11 and 25 nm Au NPs, HR-(S)TEM micrographs for precursor NPs and hybrid Au–Ag_2_Se NPs, characterization of the sample after obtaining by a reaction of Ag_2_S NPs with 25 nm Au NPs, TEM micrograph of Au–Ag_2_S hybrids obtained from Ag_2_S NRs, interplanar distances found by HRTEM analysis in Au–Ag–S samples, TEM images for reaction mechanisms study of Au–Ag–S system, theoretical relative stability of the (Ag, Au)_2_S phases, TEM and XRD characterization for different Au–Ag–S samples, zeta-potential values for hydrophilic precursor samples, and parameters for DFT calculation and corresponding results (PDF)

## References

[ref1] Hurst S. J.; Payne E. K.; Qin L.; Mirkin C. A. Multisegmented One-Dimensional Nanorods Prepared by Hard-Template Synthetic Methods. Angew. Chem., Int. Ed. 2006, 45, 2672–2692. 10.1002/anie.200504025. 16570332

[ref2] Cozzoli P. D.; Pellegrino T.; Manna L. Synthesis, Properties and Perspectives of Hybrid Nanocrystal Structures. Chem. Soc. Rev. 2006, 35, 1195–1208. 10.1039/b517790c. 17057845

[ref3] Cortie M. B.; Mcdonagh A. M. Synthesis and Optical Properties of Hybrid and Alloy Plasmonic Nanoparticles. Chem. Rev. 2011, 111, 3713–3735. 10.1021/cr1002529. 21235212

[ref4] Banin U.; Ben-Shahar Y.; Vinokurov K. Hybrid Semiconductor–Metal Nanoparticles: From Architecture to Function. Chem. Soc. Rev. 2014, 26, 97–110. 10.1021/cm402131n.

[ref5] He G.; Zhang Y.; He Q. MoS2/CdS Heterostructure for Enhanced Photoelectrochemical Performance under Visible Light. Catalysts 2019, 9, 19–21. 10.3390/catal9040379.

[ref6] Wang H.; Gao Y.; Liu J.; Li X.; Ji M.; Zhang E.; Cheng X.; Xu M.; Liu J.; Rong H.; Chen W.; Fan F.; Li C.; Zhang J. Efficient Plasmonic Au/CdSe Nanodumbbell for Photoelectrochemical Hydrogen Generation beyond Visible Region. Adv. Energy Mater. 2019, 9, 1803889 10.1002/aenm.201803889.

[ref7] Rudenko V.; Tolochko A.; Zhulai D.; Klimusheva G.; Mirnaya T.; Yaremchuk G.; Asaula V. Nonlinear Optical Properties of Metal Alkanoate Composites with Hybrid Core/Shell Nanoparticles. Appl. Nanosci. 2018, 8, 823–829. 10.1007/s13204-018-0665-4.

[ref8] Carbone L.; Cozzoli P. D. Colloidal Heterostructured Nanocrystals: Synthesis and Growth Mechanisms. Nano Today 2010, 5, 449–493. 10.1016/j.nantod.2010.08.006.

[ref9] Caro C.; Dalmases M.; Figuerola A.; García-Martín M. L.; Leal M. P. Highly Water-Stable Rare Ternary Ag-Au-Se Nanocomposites as Long Blood Circulation Time X-Ray Computed Tomography Contrast Agents. Nanoscale 2017, 9, 7242–7251. 10.1039/C7NR01110E. 28513714

[ref10] Wang Y.; Zhang P.; Mao X.; Fu W.; Liu C. Seed-Mediated Growth of Bimetallic Nanoparticles as an Effective Strategy for Sensitive Detection of Vitamin C. Sens. Actuators, B 2016, 231, 95–101. 10.1016/j.snb.2016.03.010.

[ref11] Xia Y.; Gilroy K. D.; Peng H.-C.; Xia X. Seed-Mediated Growth of Colloidal Metal Nanocrystals. Angew. Chem., Int. Ed. 2017, 56, 60–95. 10.1002/anie.201604731. 27966807

[ref12] Qiao S.; Yang Z.; Xu J.; Wang X.; Yang J.; Hou Y. Chemical Synthesis, Structure and Magnetic Properties of Co Nanorods Decorated with Fe_3_O_4_ Nanoparticles. Sci. China Mater. 2018, 61, 1614–1622. 10.1007/s40843-018-9291-y.

[ref13] Hodges J. M.; Morse J. R.; Fenton J. L.; Ackerman J. D.; Alameda L. T.; Schaak R. E. Insights into the Seeded-Growth Synthesis of Colloidal Hybrid Nanoparticles. Chem. Mater. 2017, 29, 106–119. 10.1021/acs.chemmater.6b02795.

[ref14] Burrows N. D.; Harvey S.; Idesis F. A.; Murphy C. J. Understanding the Seed-Mediated Growth of Gold Nanorods through a Fractional Factorial Design of Experiments. Langmuir 2017, 33, 1891–1907. 10.1021/acs.langmuir.6b03606. 27983861

[ref15] Kim M.; Phan V. N.; Lee K. Exploiting Nanoparticles as Precursors for Novel Nanostructure Designs and Properties. CrystEngComm 2012, 14, 7535–7548. 10.1039/c2ce25815c.

[ref16] Deshmukh S. D.; Ellis R. G.; Sutandar D. S.; Rokke D. J.; Agrawal R. Versatile Colloidal Syntheses of Metal Chalcogenide Nanoparticles from Elemental Precursors Using Amine-Thiol Chemistry. Chem. Mater. 2019, 31, 9087–9097. 10.1021/acs.chemmater.9b03401.

[ref17] Vasquez Y.; Henkes A. E.; Chris Bauer J.; Schaak R. E. Nanocrystal Conversion Chemistry: A Unified and Materials-General Strategy for the Template-Based Synthesis of Nanocrystalline Solids. J. Solid State Chem. 2008, 181, 1509–1523. 10.1016/j.jssc.2008.04.007.

[ref18] Fayette M.; Robinson R. D. Chemical Transformations of Nanomaterials for Energy Applications. J. Mater. Chem. A 2014, 2, 5965–5978. 10.1039/C3TA13982D.

[ref19] Chen X.; Fuchs H. Soft Matter Nanotechnology : From Structure to Function; Wiley-VCH Verlag GmbH & Co. KGaA: Weinheim, 2015.

[ref20] de Trizio L.; Manna L. Forging Colloidal Nanostructures via Cation Exchange Reactions. Chem. Rev. 2016, 116, 10852–10887. 10.1021/acs.chemrev.5b00739. 26891471 PMC5043423

[ref21] Park J.; Lim S.; Kwon T.; Jun M.; Oh A.; Baik H.; Lee K. Longitudinal Strain Engineering of Cu_2– *x*_S by the Juxtaposed Cu_5_FeS _4_ Phase in the Cu_5_ FeS_4_/Cu _2– *x*_ S/Cu _5_ FeS _4_ Nanosandwich. Chem. Mater. 2019, 31, 9070–9077. 10.1021/acs.chemmater.9b03342.

[ref22] Hinterding S. O. M.; Berends A. C.; Kurttepeli M.; Moret M. E.; Meeldijk J. D.; Bals S.; van der Stam W.; de Mello Donega C. Tailoring Cu^+^ for Ga^3+^ Cation Exchange in Cu_2-X_S and CuInS_2_ Nanocrystals by Controlling the Ga Precursor Chemistry. ACS Nano 2019, 13, 12880–12893. 10.1021/acsnano.9b05337. 31617701 PMC6890264

[ref23] Zhang L.; Xu L.; Zhu M.; Li C.; Li L.; Su J.; Gao Y. Pink All-Inorganic Halide Perovskite Nanocrystals with Adjustable Characteristics: Fully Reversible Cation Exchange, Improving the Stability of Dopant Emission and Light-Emitting Diode Application. J. Alloys Compd. 2019, 152913 10.1016/j.jallcom.2019.152913.

[ref24] Suri M.; Hazarika A.; Larson B. W.; Zhao Q.; Vallés-Pelarda M.; Siegler T. D.; Abney M. K.; Ferguson A. J.; Korgel B. A.; Luther J. M. Enhanced Open-Circuit Voltage of Wide-Bandgap Perovskite Photovoltaics by Using Alloyed (FA_1– *x*_ Cs_*x*_ )Pb(I_1– *x*_ Br_*x*_) _3_ Quantum Dots. ACS Energy Lett. 2019, 4, 1954–1960. 10.1021/acsenergylett.9b01030.

[ref25] Dalmases M.; Ibáñez M.; Torruella P.; Fernàndez-Altable V.; López-Conesa L.; Cadavid D.; Piveteau L.; Nachtegaal M.; Llorca J.; Ruiz-González M. L.; Estradé S.; Peiró F.; Kovalenko M. v.; Cabot A.; Figuerola A. Synthesis and Thermoelectric Properties of Noble Metal Ternary Chalcogenide Systems of Ag-Au-Se in the Forms of Alloyed Nanoparticles and Colloidal Nanoheterostructures. Chem. Mater. 2016, 28, 7017–7028. 10.1021/acs.chemmater.6b02845.

[ref26] Dalmases M.; Torruella P.; Blanco-Portals J.; Vidal A.; Lopez-Haro M.; Calvino J. J.; Estradé S.; Peiró F.; Figuerola A. Gradual Transformation of Ag_2_S to Au_2_S Nanoparticles by Sequential Cation Exchange Reactions: Binary, Ternary, and Hybrid Compositions. Chem. Mater. 2018, 30, 6893–6902. 10.1021/acs.chemmater.8b03208.

[ref27] Wang J.; Feng H.; Chen K.; Fan W.; Yang Q. Solution-Phase Catalytic Synthesis, Characterization and Growth Kinetics of Ag_2_S-CdS Matchstick-like Heteronanostructures. Dalton Trans. 2014, 43, 3990–3998. 10.1039/C3DT52693C. 24452178

[ref28] Sahu A.; Qi L.; Kang M. S.; Deng D.; Norris D. J. Facile Synthesis of Silver Chalcogenide (Ag _2_ E; E = Se, S, Te) Semiconductor Nanocrystals. J. Am. Chem. Soc. 2011, 133, 6509–6512. 10.1021/ja200012e. 21486029

[ref29] Kresse G.; Furthmüller J. Efficient Iterative Schemes for *Ab Initio* Total-Energy Calculations Using a Plane-Wave Basis Set. Phys. Rev. B 1996, 54, 11169 10.1103/PhysRevB.54.11169. 9984901

[ref30] Kresse G.; Furthmüller J. Efficiency of Ab-Initio Total Energy Calculations for Metals and Semiconductors Using a Plane-Wave Basis Set. Comput. Mater. Sci. 1996, 6, 15–50. 10.1016/0927-0256(96)00008-0. 9984901

[ref31] Kresse G.; Joubert D. From Ultrasoft Pseudopotentials to the Projector Augmented-Wave Method. Phys. Rev. B 1999, 59, 1758 10.1103/PhysRevB.59.1758.

[ref32] Blöchl P. E. Projector Augmented-Wave Method. Phys. Rev. B 1994, 50, 17953 10.1103/PhysRevB.50.17953. 9976227

[ref33] Perdew J. P.; Burke K.; Ernzerhof M. Generalized Gradient Approximation Made Simple. Phys. Rev. Lett. 1996, 77, 3865 10.1103/PhysRevLett.77.3865. 10062328

[ref34] Mikhlin Y. L.; Nasluzov V. A.; Romanchenko A. S.; Shor A. M.; Pal’Yanova G. A. XPS and DFT Studies of the Electronic Structures of AgAuS and Ag_3_AuS_2_. J. Alloys Compd. 2014, 617, 314–321. 10.1016/j.jallcom.2014.08.014.

[ref35] Krukau A. V.; Vydrov O. A.; Izmaylov A. F.; Scuseria G. E. Influence of the Exchange Screening Parameter on the Performance of Screened Hybrid Functionals. J. Chem. Phys. 2006, 125, 224106 10.1063/1.2404663. 17176133

[ref36] Sharma R. C.; Chang Y. A. The Ag–S (Silver-Sulfur) System. Bull. Alloy Phase Diagr. 1986, 7, 263–269. 10.1007/BF02869003.

[ref37] Wang J.; Fan W.; Yang J.; Da Z.; Yang X.; Chen K.; Yu H.; Cheng X. Tetragonal - Orthorhombic - Cubic Phase Transitions in Ag_2_Se Nanocrystals. Chem. Mater. 2014, 26, 5647–5653. 10.1021/cm502317g.

[ref38] Schoen D. T.; Xie C.; Cui Y. Electrical Switching and Phase Transformation in Silver Selenide Nanowires. J. Am. Chem. Soc. 2007, 129, 4116–4117. 10.1021/ja068365s. 17367137

[ref39] Kalsin A. M.; Fialkowski M.; Paszewski M.; Smoukov S. K.; Bishop K. J. M.; Grzybowski B. A. Electrostatic Self-Assembly of Binary Nanoparticle Crystals with a Diamond-like Lattice. Science 2006, 312, 420–424. 10.1126/science.1125124. 16497885

[ref40] Huang Z.; Zhao Z. J.; Zhang Q.; Han L.; Jiang X.; Li C.; Cardenas M. T. P.; Huang P.; Yin J. J.; Luo J.; Gong J.; Nie Z. A Welding Phenomenon of Dissimilar Nanoparticles in Dispersion. Nat. Commun. 2019, 10, 1–8. 30644406 10.1038/s41467-018-08206-6 PMC6333817

[ref41] Xia X.; Wang Y.; Ruditskiy A.; Xia Y. 25th Anniversary Article: Galvanic Replacement: A Simple and Versatile Route to Hollow Nanostructures with Tunable and Well-Controlled Properties. Adv. Mater. 2013, 25, 6313–6333. 10.1002/adma.201302820. 24027074

[ref42] Pattadar D. K.; Masitas R. A.; Stachurski C. D.; Cliffel D. E.; Zamborini F. P. Reversing the Thermodynamics of Galvanic Replacement Reactions by Decreasing the Size of Gold Nanoparticles. J. Am. Chem. Soc. 2020, 142, 19268–19277. 10.1021/jacs.0c09426. 33140961

[ref43] van Huis M. A.; Figuerola A.; Fang C.; Béché A.; Zandbergen H. W.; Manna L. Chemical Transformation of Au-Tipped CdS Nanorods into AuS/Cd Core/Shell Particles by Electron Beam Irradiation. Nano Lett. 2011, 11, 4555–4561. 10.1021/nl2030823. 21995508

