# Supplementary material for: Spontaneous Hetero-attachment of Single-Component Colloidal Precursors for the Synthesis of Asymmetric Au–Ag2X (X = S, Se) Heterodimers
Source: Chem Mater. 2022 Dec 12;34(24):10849–60. doi: 10.1021/acs.chemmater.2c01838 (PMC9799023; doi:10.1021/acs.chemmater.2c01838)
Supplement: Supplementary file 1 — cm2c01838_si_001.pdf [file cm2c01838_si_001.pdf]

Supporting Information

Spontaneous Heteroattachment of Single Component Colloidal Precursors for the Synthesis of Asymmetric Au-Ag_2X (X = S, Se) Heterodimers

Mengxi Lin[1,2], Guillem Montana [1,2 †], Javier Blanco[2,3], Lluís Yedra[2,3], Heleen van Gog[4], Marijn A. van Huis[5], Miguel López-Haro[6], José Juan Calvino[6], Sònia Estradé[2,3], Francesca Peiró[2,3], Albert Figuerola*[1,2].

1 Department of Inorganic and Organic Chemistry, Inorganic Chemistry Section, University of Barcelona, Carrer de Martí i Franquès, 1-11, 08028 Barcelona, Spain

2 Institute of Nanoscience and Nanotechnology, University of Barcelona, Carrer de Martí i Franquès, 1-11, 08028 Barcelona, Spain

3 Laboratory of Electron Nanoscopies (LENS-MIND), Department of Electronics and Biomedical Engineering, Universitat de Barcelona,C/Martí I Franquès 1 08028, Barcelona, Spain

4 Nanostructured Materials and Interfaces, Zernike Institute for Advanced Materials, University of Groningen, Nijenborgh 4, 9747 AG Groningen, The Netherlands

5 Soft Condensed Matter, Debye Institute for Nanomaterials Science, Utrecht University, Princetonplein 5, 3584 CC Utrecht, The Netherlands

6 Departamento de Ciencia de los Materiales e Ingeniería Metalúrgica y Química Inorgánica, Facultad de Ciencias, Universidad de Cádiz, Cádiz, 11510, Spain

Supporting Experiments

Synthesis of 11 nm and 25 nm Au NPs

1.7 g (6.4 mmol, 2.9 ml) of oleylamine was added into 49 ml of toluene and the whole mixture was heated to boil, followed by a rapid injection of 50 mg (0.15 mmol) of tetrachloroauric acid in 1.0 g (3.7 mmol, 1.2 ml) of oleylamine and 1.0 ml of toluene. The reaction mixture was heated under reflux for 16 mins for 11nm Au NPs, and 2h for 25 nm Au NPs. The color change from yellow to red was started to be observed from 5 mins. Afterwards, the reaction mixture was cooled down to room temperature and washed three times with EtOH by centrifugation. The final product was dispersed in 1mL toluene.

Supporting Figures

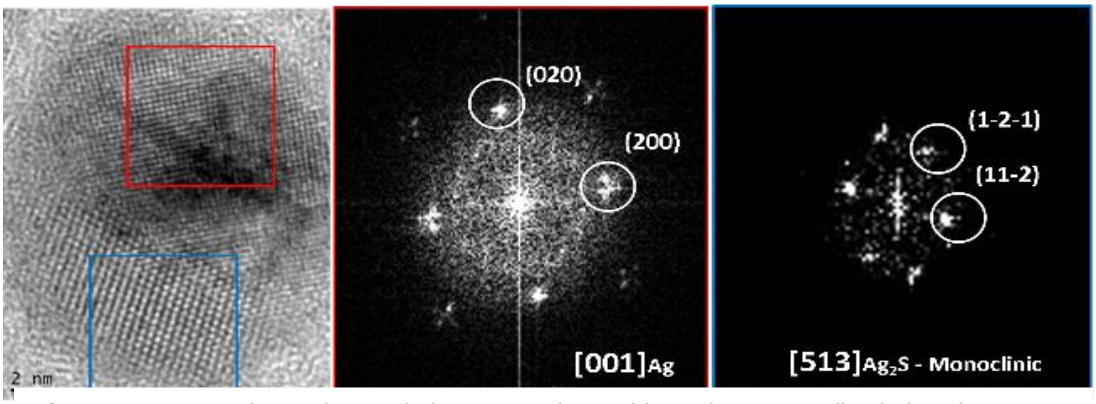

Figure S1. HRTEM image from a dark-contrasted *acanthite* and corresponding indexed FFT.

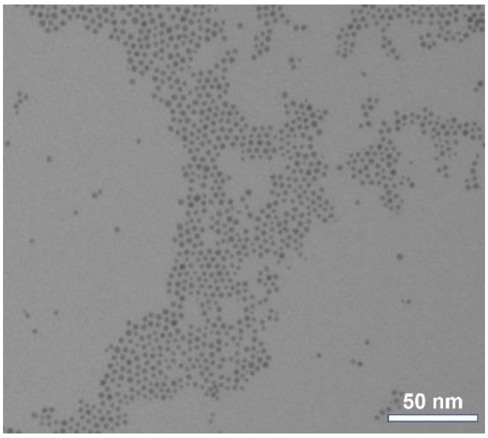

Figure S2. TEM image of Au NPs.

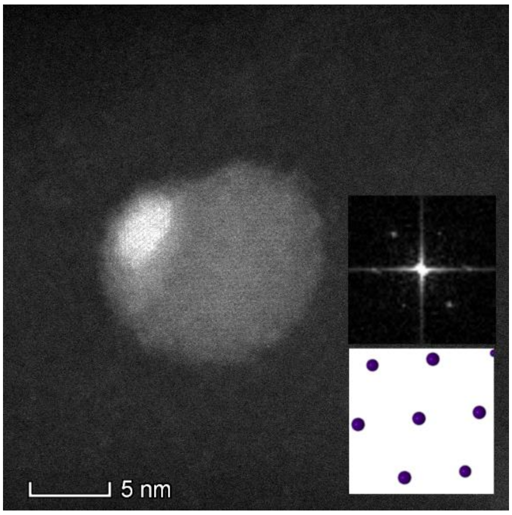

Figure S3: HR-STEM image of a Au-Ag_2Se particle with a FFT of the Au bright part that could be indexed as [101] oriented gold (see theoretical diffraction below the FFT).

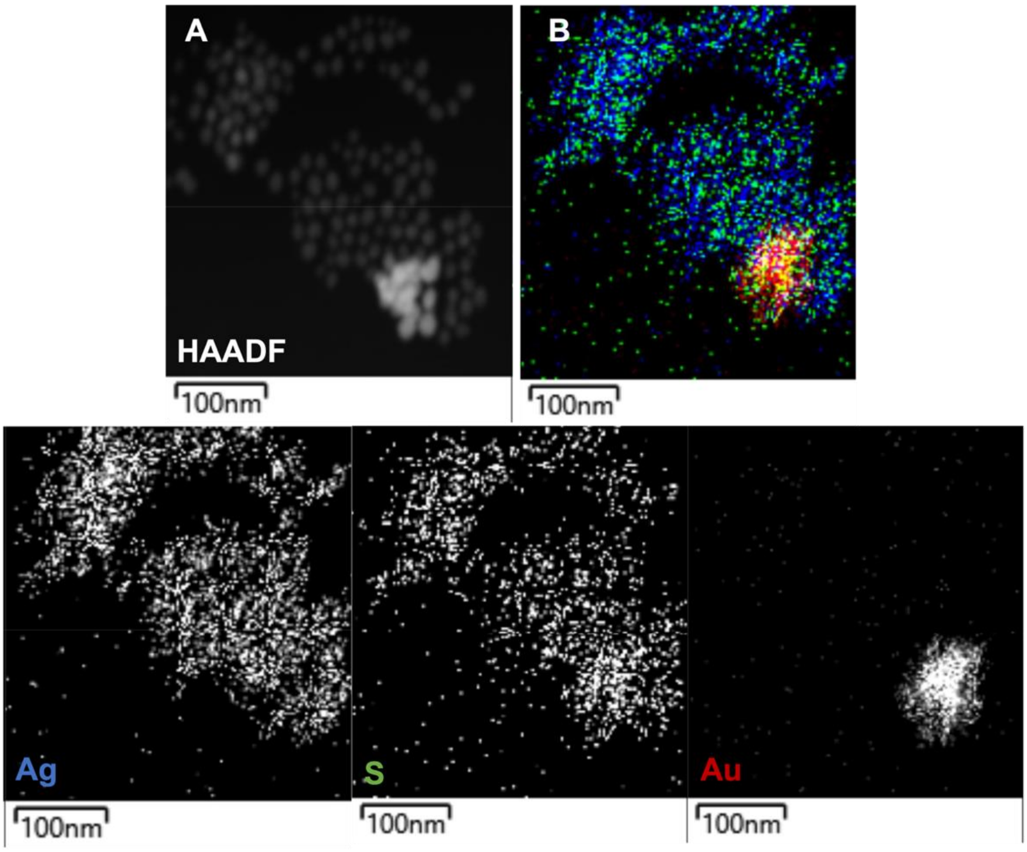

Figure S4: (A) HAADF image after reaction of Ag_2S NPs with 25 nm Au NPs, (B) Elemental mappings extracted from XEDS spectra.

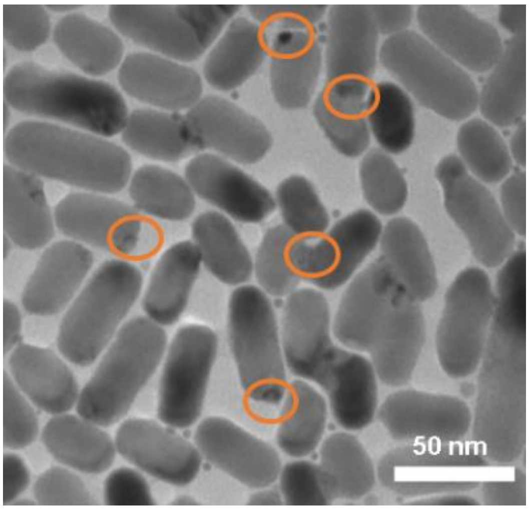

Figure S5: TEM image after reaction of Ag_2S NRs with 3.5 nm Au NPs.

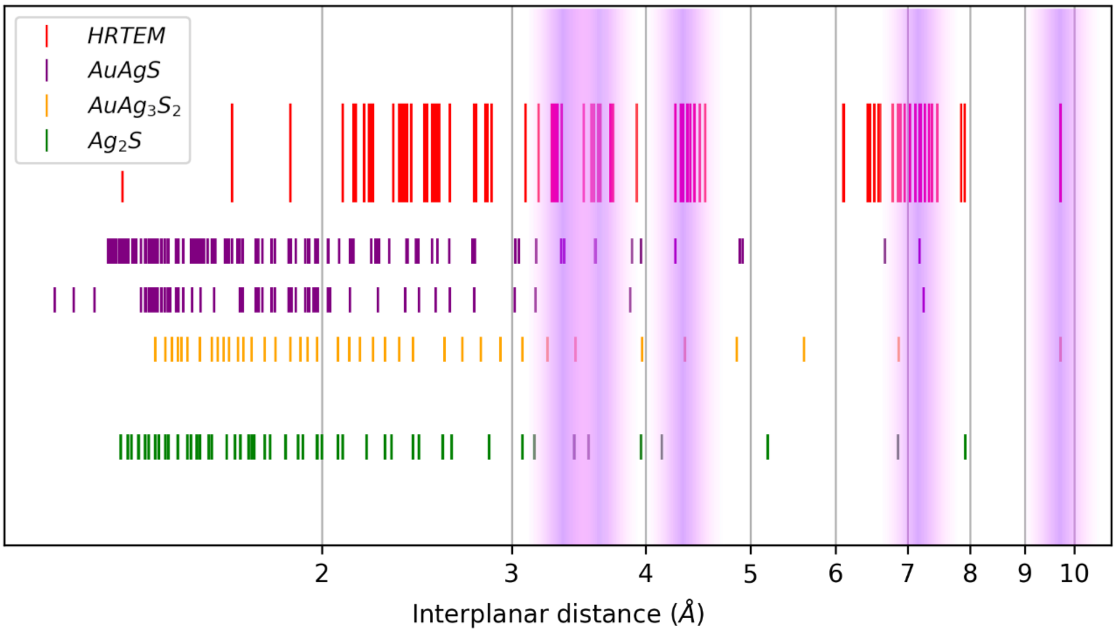

Figure S6: The measured interplanar distances in 14 HRTEM images of Au-Ag-S samples (in red) in comparison with the interplanar distances from reference patterns (AuAgS in purple, Ag_2S in green, and $AuAg_3S_2$ in yellow) in logarithmic scale. The shaded areas in the graph show interplanar distances that can unequivocally be attributed to one of the ternary compounds. Y axis is arbitrary to separate the different samples and references.

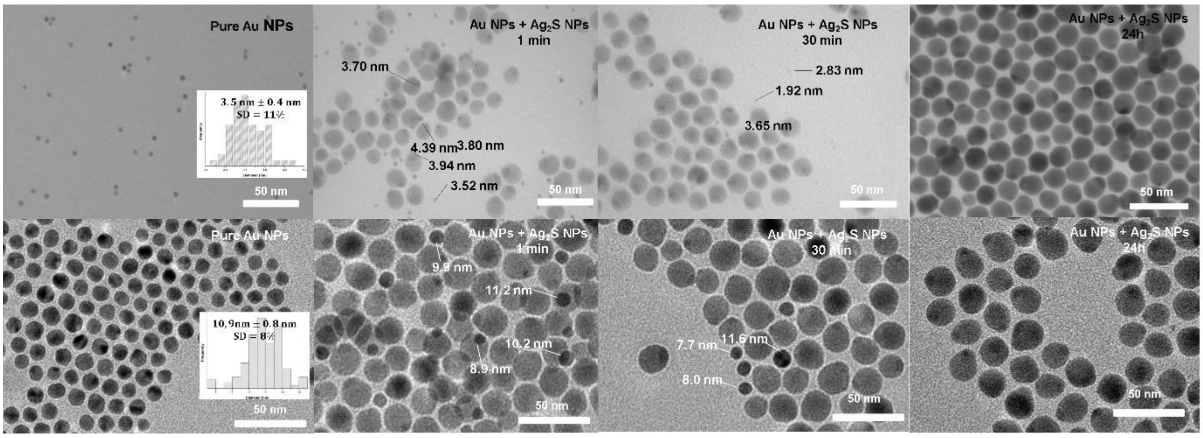

Figure S7: TEM images of added Au-precursors and aliquots at specific time along the reaction of mixing Ag_2S NPs with 2 different sized Au NPs: top: 3.5 nm Au NPs, bottom: 11 nm Au NPs.

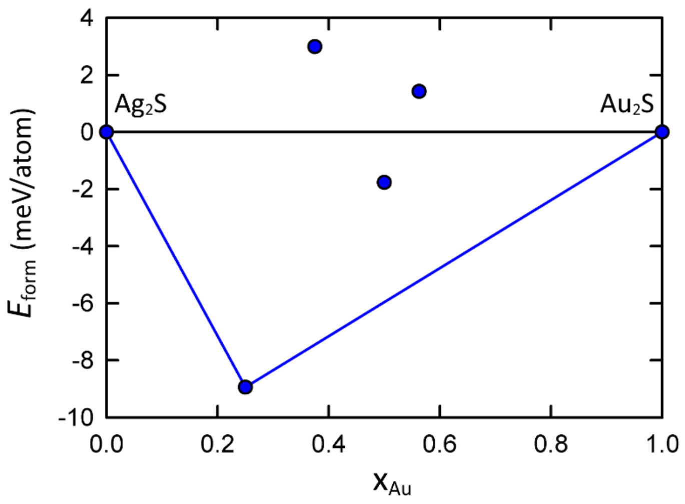

Figure S8. Relative stability of the (Ag,Au)$_2$S phases evaluated with respect to Ag_2S and Au_2S, calculated using GGA-PBE using Eq. (2) in the main text. The numerical values are listed in Table S2. The blue line is the common tangent line connecting the energetically most favorable phases.

Supporting Tables

Table. S1: The TEM and corresponding XRD for the following samples.

<table>
<tr><th>TEM</th><th>XRD</th></tr>
<tr><td>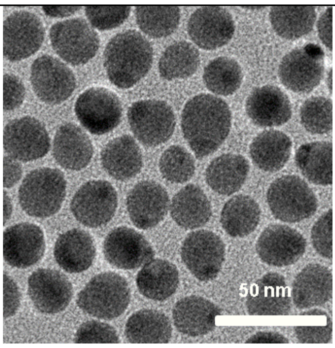

Ag_2S + **11nm** Au (toluene)</td><td>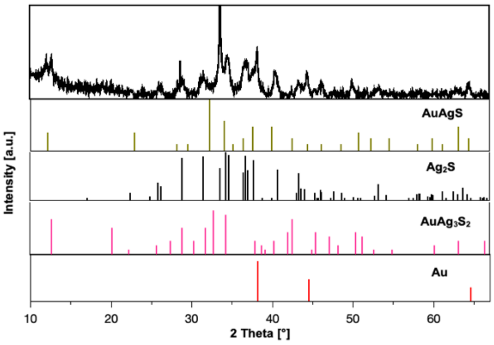
</td></tr>
<tr><td>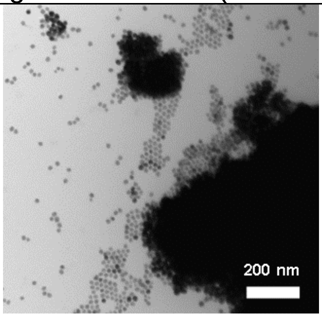

Ag_2S + **25 nm** Au (toluene)</td><td>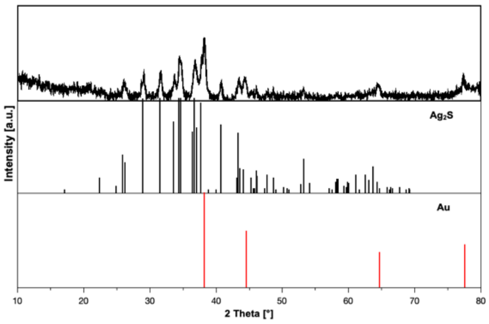
</td></tr>
<tr><td>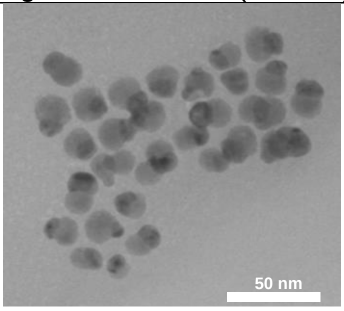

Ag_2S + 3.5 nm Au (**water**)</td><td>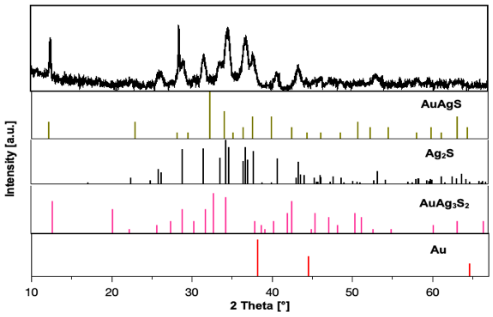
</td></tr>
</table>

Table. S2: Zeta-potential for the following samples

Sample	Zeta-potential [mV]
Au-MUA in water	-62.1
Ag_2S-MUA in water	-59.6

Table S3. Overview of M–S, M–Se, M, and M–M (M=Au,Ag) phases considered for the DFT calculations in this work, along with the used *k*-mesh and calculated lattice parameters as obtained after full relaxation with GGA-PBE.

System	Compound	Atoms per unit cell	*k*-mesh	Lattice parameters
M–Se	Ag_2Se	12	6×8×8	*a*=4.526 Å, *b*=7.080 Å, *c*=7.690 Å
	Ag_3AuSe_2	48	4×4×4	*a*=10.109 Å
M–S	Ag_2S	12	10×6×6	*a*=4.357 Å, *b*=7.924 Å, *c*=8.713 Å, β=116.2°
	Ag_3AuS_2	18	6×6×6	*a*=7.910 Å, *c*=8.738 Å, γ=120.0°
	$Ag_{30}Au_{18}S_{24}$	72	3×3×4	*a*=*b*=13.922 Å, *c*=9.011 Å, α=90.1°,β=89.9°, γ=119.8°
	AgAuS	72	3×3×4	*a*=*b*=13.815 Å, *c*=9.215 Å, α=90.1°,β=89.9°, γ=119.8°
	$Ag_{21}Au_{27}S_{24}$	72	3×3×4	*a*=*b*=13.918 Å, *c*=9.127 Å, α=90.0°,β=90.0°, γ=120.0°
	Au_2S	6	8×8×8	*a*=5.324 Å
M	Ag	4	28×28×28	*a*=4.162 Å
	Au	4	28×28×28	*a*=4.156 Å
M–M	Ag_3Au_1	4	28×28×28	*a*=4.144 Å
	Ag_2Au_2	4	28×28×28	*a*=*b*=4.113 Å, *c*=4.208 Å
	Ag_1Au_3	4	28×28×28	*a*=4.149 Å

Table S4. Formation energies of the $(Ag_{1-x}Au_x)_2S$ phases evaluated with respect to Ag_2S and Au_2S, calculated with GGA-PBE using Eq. (2) in the main text.

Phase	Trivial name	x_{Au}	E_{form} (meV/atom)
Ag_2S		0.00	0.0
Ag_3AuS_2	uytenbogaardite	0.25	−8.9
$Ag_{30}Au_{18}S_{24}$	petrovskaite	0.375	3.0
AgAuS	petrovskaite	0.50	−1.8
$Ag_{21}Au_{27}S_{24}$	petrovskaite	0.563	1.4
Au_2S		1.00	0.0
